# Reproducible floating car data monitoring for data-scarce highway corridors: A TomTom–HERE benchmark study

**DOI:** 10.1371/journal.pone.0357158

**Published:** 2026-08-31

**Authors:** Wenjie Sun, Safizahanin binti Mokhtar, Ruiqing Li, Zhou Zou

**Affiliations:** 1 Department of Urban and Regional Planning, Universiti Teknologi Malaysia, Skudai, Johor, Malaysia; 2 Faculty of Computing, Universiti Teknologi Malaysia, UTM Johor Bahru, Johor, Malaysia; 3 School of Physics and Electronic Information Engineering, Hubei Engineering University, Xiaogan, P. R. China; Khalifa University, UNITED ARAB EMIRATES

## Abstract

Data-scarce highway corridors require continuous monitoring, but fixed detectors are often sparse, costly, and difficult to maintain. This study developed a reproducible floating car data workflow for corridor traffic monitoring. The workflow includes scheduled data collection, rule-based filtering, database storage, indicator calculation, cross-platform comparison, and result export. The case study focused on the Kuching–Telok Melano section of the Pan Borneo Highway in Sarawak, Malaysia. A 42-point TomTom virtual monitoring panel was collected at 15 min intervals from 8 December 2025–28 February 2026. HERE traffic records were used as a 30 min external benchmark during the overlapping period from 8 to 31 December 2025 after temporal and spatial harmonization. The TomTom panel achieved 97.30% mean daily availability and detected a repeated evening pressure window near 19:30. The TomTom–HERE comparison showed similar traffic-pressure patterns at the matched block–time scale. These findings suggest that platform FCD can support corridor monitoring where fixed detectors are limited, but they should not be read as ground-truth validation.

## Introduction

Long-distance highway corridors require continuous traffic monitoring because local operational changes can affect the performance of the whole corridor. These corridors often serve commuting, freight, tourism, emergency access, and regional travel. When one section experiences delay, the effect may extend beyond a local bottleneck because alternative routes are limited [1]. This problem is more severe in data-scarce highway corridors. In this study, a data-scarce highway corridor is defined as a long-distance road corridor where continuous fixed-detector coverage, publicly available traffic counts, calibrated travel-time surveys, and open traffic-state records are limited or absent along substantial parts of the route. This does not mean that no traffic information is available. It means that the available information is not sufficient for continuous, corridor-wide, and repeatable traffic-state monitoring.

Traditional traffic detection systems provide useful evidence, but continuous deployment on long corridors is costly and operationally difficult. Inductive loops, Bluetooth readers, and traffic cameras can measure local traffic conditions, yet they require field installation, power supply, maintenance, and communication infrastructure. Manual travel-time surveys can support short-term field studies, but they cannot describe repeated daily variation or long-term operating patterns. Therefore, data-scarce corridors require a lower-cost, repeatable, and scalable supplementary monitoring method [1].

Platform floating car data (FCD) provide a practical basis for virtual traffic detection. Recent reviews show that FCD can support speed monitoring, travel-time estimation, congestion detection, and traffic-state inference across different spatial scales [2]. However, commercial FCD are sampled and processed by vendors. Their reliability depends on penetration rate, spatial coverage, temporal coverage, map matching, provider-specific aggregation rules, and the stability of platform responses [3–6]. These characteristics make FCD useful for corridor monitoring, but they also require quality control, repeatable processing, and careful interpretation.

Previous studies have used FCD, probe-vehicle data, Bluetooth data, and stationary detectors to estimate speeds, travel times, traffic volumes, and traffic states [7–9]. These studies show the analytical value of new traffic data sources. However, three issues remain important when platform FCD are used as a road traffic monitoring system rather than as a one-time analytical dataset. First, many studies report traffic indicators but provide limited detail on automated acquisition, filtering, storage, and audit procedures. Second, the design of fixed virtual monitoring points is often less explicit than the final congestion results, although the monitoring-point structure determines the spatial detection logic. Third, cross-platform agreement does not establish absolute accuracy because vendors may differ in probe populations, map matching, spatial units, update intervals, confidence rules, and aggregation procedures.

To address these gaps, this study develops a script-based FCD workflow for continuous corridor monitoring. The empirical case is the Kuching–Telok Melano section of the Pan Borneo Highway in Sarawak, Malaysia. The study uses 42 fixed-coordinate TomTom virtual monitoring points as the main detection panel and HERE traffic records as an external benchmark signal during the overlapping benchmark period. The workflow has six modules: input configuration, scheduled data collection, rule-based filtering, database storage, indicator generation, and cross-platform comparison. It does not present a general-purpose commercial software package. Instead, it shows how platform FCD can be collected, cleaned, stored, and compared for corridor monitoring.

This study addresses three research questions. First, can a fixed-point platform FCD panel provide stable daily acquisition coverage for a data-scarce highway corridor? Second, can the panel detect interpretable intraday traffic-state patterns from speed and travel-time indicators? Third, do TomTom-derived pressure indicators converge with HERE benchmark signals after temporal and spatial harmonization during the overlapping benchmark period?

Unlike studies that begin with an already assembled FCD dataset and proceed directly to traffic analysis, this study specified and operated the monitoring process before the final analytical dataset was produced. The fixed coordinates, acquisition schedules, response-field rules, missing-record logs, storage structure, spatial harmonization, and reproducible exports were treated as components of the research design. The study therefore evaluates both the operation of the data-producing system and the traffic patterns derived from it. Specifically, the workflow repeatedly queries the same 42 fixed anchors, treats daily availability and missing-interval diagnostics as system outputs, links acquisition through benchmark export in one auditable chain, and compares the two platforms only after common block–time harmonization.

The rest of the paper is organized as follows. The Materials and methods section describes the study corridor, platform data sources, system framework, cleaning model, indicator definitions, and benchmark calculation module. The Results section reports system availability, traffic-state detection results, and TomTom–HERE benchmark consistency. The Discussion section discusses system reliability, external consistency, scalability, deployment value, and evidence boundaries. The Conclusion section summarizes the main findings and future validation needs.

## Materials and methods

### Study corridor and workflow framework

**Fig 1:**
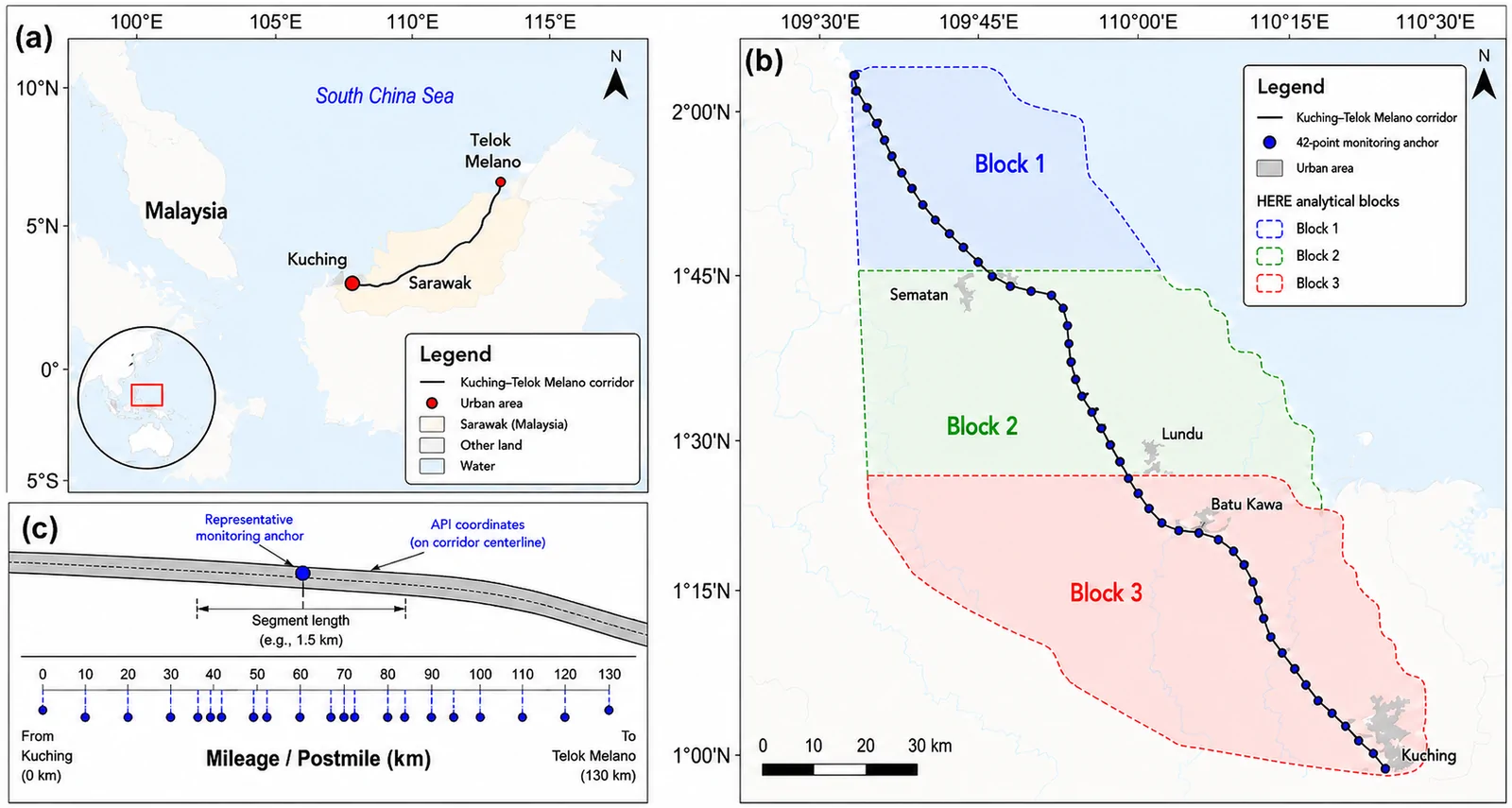
Pan Borneo Highway case corridor, TomTom virtual monitoring points, and HERE benchmark blocks. *Note.* Panels A and B were drawn using public-domain Natural Earth basemap vector layers [10]. The corridor line, monitoring points, benchmark-block boundaries, labels, and panel C schematic were generated by the authors from the study coordinate configuration and analytical design. No Google Maps, Google Earth, proprietary satellite imagery, or proprietary web-map tiles were used.

The case corridor is the Kuching–Telok Melano section of the Pan Borneo Highway in Sarawak, Malaysia. The monitored length is about 130 km. The corridor connects the urban fringe of Kuching with western coastal settlements, including Lundu, Sematan, and Telok Melano. It is a long-distance linear corridor with mixed commuting, town-node, coastal-settlement, tourism, freight, and regional travel functions. Fixed traffic-sensing facilities and public traffic-state records are limited along substantial parts of the corridor, making it suitable for testing a data-scarce highway-monitoring workflow. The spatial layout of the case corridor, the 42 TomTom virtual monitoring points, and the three HERE benchmark blocks is shown in Fig 1.

The 42 TomTom virtual monitoring points were selected to provide repeatable longitudinal coverage along the approximately 130 km corridor. They were not designed as a strictly equally spaced statistical sample. Instead, the locations were distributed to represent the Kuching–Batu Kawa urban-fringe section, the Batu Kawa–Lundu–Sematan intermediate section, and the Sematan–Telok Melano coastal section, while maintaining continuous coverage between the main corridor locations. Candidate coordinates were checked against the intended highway alignment, and locations susceptible to ambiguous matching with nearby non-corridor roads, minor access roads, parallel roads, or complex junctions were avoided or adjusted before the final monitoring coordinates were fixed.

The monitoring panel shown in Fig 1 was designed to convert platform FCD requests into a repeated corridor detection structure. Each TomTom monitoring point is a fixed latitude–longitude anchor used for scheduled traffic-flow acquisition. After the spatial coverage and road-matching checks, the final 42 coordinates were retained as stable monitoring locations throughout the study period. The panel was therefore designed for stable repeated monitoring rather than random spatial sampling. This design reduces the risk of ad hoc sampling, repeated manual point selection, and uncontrolled spatial gaps. It also allows the same point–time structure to be used for availability assessment, traffic-state indicator generation, and later aggregation to benchmark blocks.

The HERE benchmark design used three larger corridor blocks rather than point-level matching. This was necessary because TomTom and HERE do not return identical spatial units. TomTom records were requested at fixed coordinate anchors and returned traffic-flow information for the closest road fragment. HERE records were queried and summarized by benchmark areas and returned road-segment traffic states. Therefore, the two platforms were not compared at the raw coordinate or raw road-segment level. Instead, both sources were harmonized to common block–time records before consistency analysis. The point-to-block assignment used for this harmonization is summarized in Table 1. The complete virtual monitoring point and benchmark-block configuration is provided in S1 Table.

**Table 1:** Geographic coverage, approximate corridor length, HERE bounding-box coordinates, and TomTom point assignments of the benchmark blocks.

Block	Approximate geographic coverage	Approx. length	HERE bounding box	Assigned TomTom point IDs	Number of points
Block 1	Sematan–Telok Melano coastal section	25 km	Latitude: 1.3800–2.0052°N; longitude: 109.6353–109.8685°E	A036, A037, A038, A039, A040, A041, A042	7
Block 2	Batu Kawa–Lundu–Sematan intermediate section	45 km	Latitude: 1.3800–2.0052°N; longitude: 109.8685–110.1016°E	A001, A002, A003, A004, A008, A009, A013, A017, A019, A020, A022, A023, A024	13
Block 3	Kuching–Batu Kawa urban-fringe section	60 km	Latitude: 1.3800–2.0052°N; longitude: 110.1016–110.3347°E	A005, A006, A007, A010, A011, A012, A014, A015, A016, A018, A021, A025, A026, A027, A028, A029, A030, A031, A032, A033, A034, A035	22

*Note.* HERE coordinates define the benchmark query areas, whereas TomTom point IDs denote fixed monitoring anchors used for repeated 15 min acquisition. Both sources were aggregated to common 30 min block–time records. Full spatial coordinates and point-to-block metadata are provided in the reproducibility package.

The three benchmark blocks correspond to the corridor sections illustrated in Fig 1. Block 1 represents the approximately 25 km Sematan–Telok Melano coastal section, Block 2 represents the approximately 45 km Batu Kawa–Lundu–Sematan intermediate section, and Block 3 represents the approximately 60 km Kuching–Batu Kawa urban-fringe section. Together, the three blocks describe the approximately 130 km study corridor. All corridor alignments and monitoring-point locations were manually reviewed in QGIS 3.22.4 with reference to OpenStreetMap road data [11,12]. The road alignments and point locations were adjusted where necessary to maintain consistency with the intended highway corridor, reduce ambiguous matching with nearby roads or junctions, and improve the reliability of section-length calculation and block assignment. The bounding-box coordinates define the HERE query areas, whereas the assigned TomTom point IDs define the fixed monitoring records aggregated within each block. The comparison is therefore performed at the common block–time level rather than at the raw coordinate or individual road-segment level.

The case corridor is suitable for testing the proposed method. Development intensity and geographic conditions vary along the route. Demand differs between the Kuching urban fringe, intermediate town nodes, and coastal settlements. These conditions create a need for repeated monitoring, but traditional section-based sensing is difficult to scale continuously over the whole corridor. This corridor was therefore used to test whether scheduled data collection, rule-based cleaning, database storage, indicator generation, and cross-platform comparison can support monitoring under data-scarce conditions.

**Fig 2:**
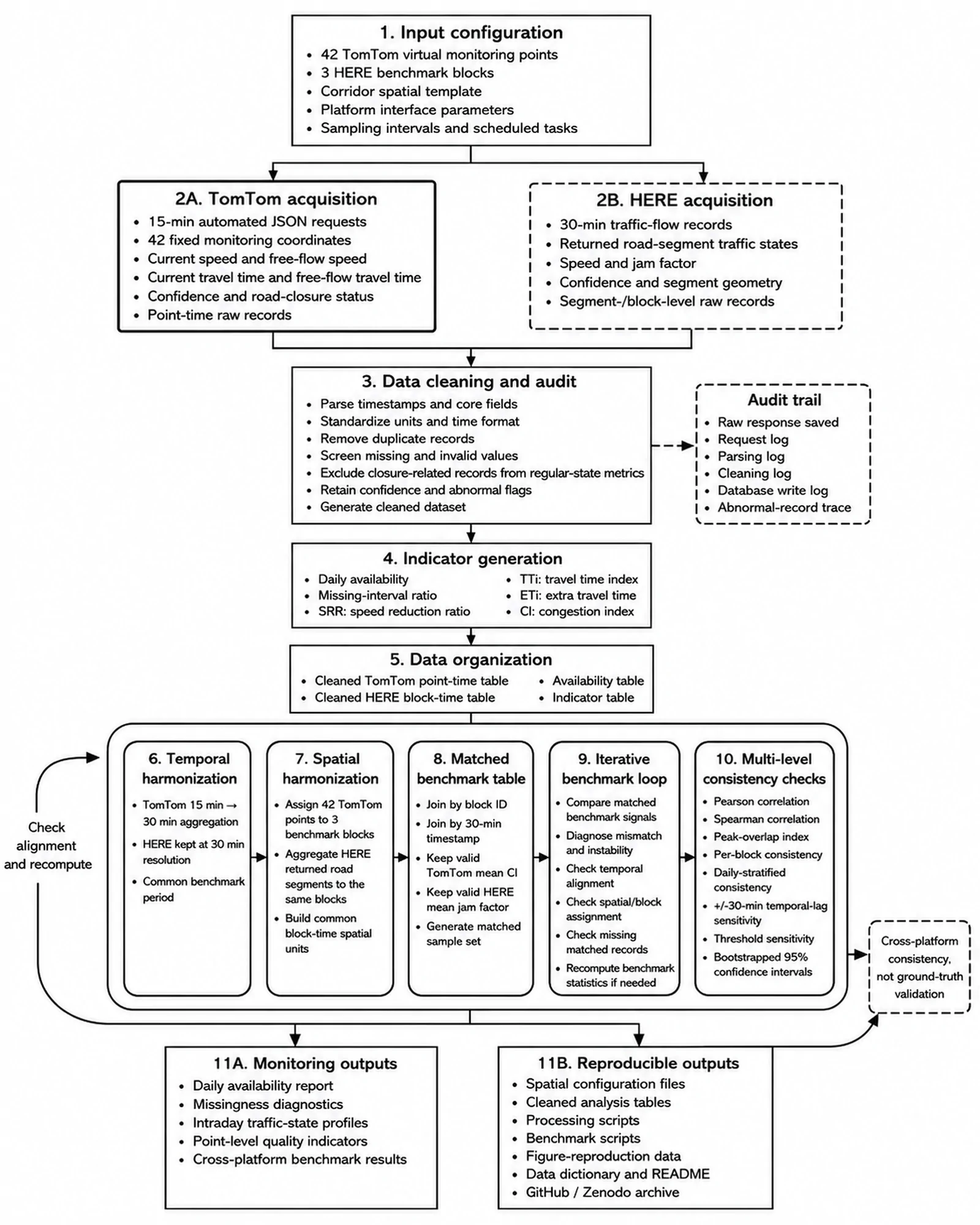
TomTom–HERE FCD workflow for reproducible corridor monitoring. *Note.* TomTom is the 15 min monitoring panel, and HERE is the 30 min benchmark source. Comparison is performed after temporal, spatial, and block–time harmonization. FCD = floating car data; CI = congestion index; TTI = travel time index.

The proposed workflow was designed to build a stable corridor traffic-data process. It collects platform traffic-state data, identifies invalid records, stores point-level records, and uses HERE data for comparison. Fig 2 summarizes the steps from data collection to cleaning, indicator calculation, benchmark matching, and export.

As shown in Fig 2, the input-configuration layer defines the spatial template, including the 42 TomTom virtual monitoring points, three HERE benchmark blocks, platform request parameters, sampling intervals, and scheduled tasks. This layer provides a common spatial reference for acquisition, quality control, aggregation, and cross-platform comparison. The acquisition layer connects to TomTom and HERE platform traffic services. The TomTom branch sends scheduled requests at 15 min intervals for the fixed monitoring points, parses the returned traffic-flow fields, applies quality control, computes traffic indicators, and stores point–time records. The HERE branch obtains traffic-flow records at 30 min resolution for the benchmark areas, parses returned road-segment traffic states, applies quality control, and generates block-level benchmark records.

The two platforms are not compared at the raw interface level. TomTom 15 min records are first aggregated to 30 min periods. TomTom points and HERE returned road segments are then aggregated to the same three benchmark blocks. The final matched benchmark table is constructed by joining records with the same benchmark block and 30 min period. This design avoids assuming that the two platforms have identical spatial units or identical measurement scales.

Fig 2 shows how data collection, quality control, storage, indicator calculation, benchmark matching, and export are combined. Internal stability is evaluated through data availability and missing-interval diagnostics, traffic-state utility through speed, travel-time, CI, and TTI profiles, and cross-platform consistency through matched TomTom–HERE block–time indicators.

### Data sources, experimental environment, and storage audit

The data sources include two platform FCD services. TomTom traffic flow data form the main virtual detection panel, while HERE traffic flow data provide an external comparison signal during the overlapping benchmark period. Both sources are vendor-processed traffic-state services rather than field-detector measurements. The TomTom Flow Segment Data service provides speeds and travel times for the road fragment closest to requested coordinates [13]. The HERE Traffic API provides traffic-flow information such as speed, jam factor, and segment geometry for user-defined areas [14]. Table 2 summarizes the two platform data sources, spatial units, key variables, and harmonization rules used in the monitoring and benchmark workflow.

**Table 2:** Data sources, spatial units, variables, and harmonization rules.

Source	Role	Period	Raw temporal resolution	Spatial unit	Main variables	Harmonization rule
TomTom	Main virtual detection source	8 Dec 2025–28 Feb 2026	15 min	42 fixed virtual monitoring points	Current speed, free-flow speed, current travel time, free-flow travel time, confidence, road-closure status	Stored as point–time records; aggregated to 30 min only for HERE comparison
HERE	External benchmark source	8–31 Dec 2025	30 min	Three benchmark blocks and returned road segments	Speed, jam factor, confidence, segment geometry	Aggregated to block–time records and matched with TomTom block-level indicators

*Note.* CI = congestion index; TTI = travel time index. TomTom records are aggregated to 30 min only during the HERE comparison stage. The one-month HERE sample was collected for the external benchmark module and was used only within the overlapping benchmark window.

The collection and analysis procedures were designed to comply with the applicable terms and conditions of the TomTom and HERE data sources at the time of data collection. The study used authorized developer/API access to obtain platform traffic-flow records for academic corridor-monitoring analysis. API keys, account credentials, access tokens, private request headers, full key-bearing request URLs, complete raw API dumps, full vendor-returned road geometries, local database dumps, and private source-data folders are not redistributed. The public release contains cleaned, derived, aggregated, and de-identified tables, spatial metadata, data dictionaries, configuration templates, processing scripts, benchmark scripts, figure-reproduction data, and representative API-workflow documentation needed to reproduce the reported results.

TomTom data support point-level traffic-state monitoring. The study places 42 fixed virtual monitoring points on the Kuching–Telok Melano corridor. Data are collected from 8 December 2025–28 February 2026 at a raw interval of 15 min. The main variables are current speed, free-flow speed, current travel time, free-flow travel time, confidence, road-closure status, timestamp, and returned road-segment information. Speed is measured in km/h, and travel time is measured in seconds. TomTom records are first organized as point–time records to describe continuous changes along the corridor. They are aggregated to 30 min block–time records only when the TomTom–HERE benchmark is calculated.

HERE data are used for external benchmark assessment. For the external benchmark module, a one-month HERE sample was collected from 8 to 31 December 2025 to test cross-platform consistency during a clearly overlapping period with the TomTom monitoring panel. HERE records are kept at 30 min resolution and are aggregated to three corridor benchmark blocks using road-segment geometry and location. The main variables are speed, jam factor, confidence, timestamp, and segment geometry. HERE speed is measured in km/h, and jam factor is treated as a dimensionless congestion signal. HERE is used as an external platform reference after temporal and spatial harmonization.

The public package provides the metadata and code needed to check the data-collection design and reproduce the cleaned tables. It includes the TomTom point-to-block assignment table, the HERE benchmark-block coordinate definition table, configuration templates, de-identified acquisition templates, processing scripts, figure-reproduction data, and benchmark outputs. The acquisition templates document the platform request workflow, but they require user-supplied TomTom and HERE developer credentials. This release documents the workflow without sharing restricted platform materials.

The acquisition module sends requests to platform traffic services at fixed task intervals and saves raw responses locally before any cleaning step. This keeps a record of each acquisition step. If a cleaning result is abnormal, the raw response example, request log, parsing record, and database write log can be checked to identify whether the issue comes from the platform response, network request, field parsing, cache access, database writing, or later processing. Cleaned data are organized into two core tables: the TomTom point–time table and the HERE block–time table. The former supports continuous traffic-state monitoring, and the latter supports cross-platform benchmarking.

The acquisition and analysis pipeline was implemented on a standard local workstation. Java and Spring Boot managed scheduled acquisition, PostgreSQL stored cleaned records and audit logs, and Python with pandas and NumPy supported cleaning, aggregation, indicator calculation, and figure reproduction [15–18]. Detailed hardware specifications, software versions, cache implementation, and task-level logging fields are provided in S1 Text.

### Ethics statement

This study used aggregated traffic-state information from platform traffic services. It did not involve human participants, interviews, surveys, personal identifiers, individual trajectory tracking, or animal subjects. All analyses were conducted at the corridor, point–time, and block–time scales. No individual traveler can be identified from the data used in this study. Formal human-subject ethics approval was not required. No physical field sampling, roadside installation, land access, protected-area access, or protected-species sampling was conducted; therefore, no field permit was required.

### Data cleaning, indicator generation, and benchmark calculation

Platform traffic-flow records require cleaning before corridor traffic analysis because they are vendor-processed records, not direct detector measurements. Returned records may include inconsistent timestamps, duplicate responses, missing core fields, non-positive speed or travel-time values, low-confidence fields, road-closure flags, and spatial-unit differences between platforms. The analysis therefore includes field parsing, validity checks, indicator calculation, temporal matching, spatial matching, matched-sample construction, and benchmark calculation. Table 3 summarizes the full workflow.

**Table 3:** Integrated data cleaning, indicator generation, and benchmark workflow.

Stage	Step	Procedure	Main output	Formula/source
Data input	1	Load raw platform traffic-flow records ℛ, including TomTom point-level records and HERE block-level records.	Raw platform record set ℛ	Platform fields [13,14]
Field parsing and unit control	2	Parse timestamp, point ID, road-segment ID, speed, travel time, confidence, and road-closure status. Standardize speed to km/h and travel time to seconds.	Structured and unit-consistent records	Platform response definitions [13,14]
Time processing	3	Convert timestamps to a unified local time format and generate date *d*, 15 min TomTom interval *t*, and 30 min benchmark period τ.	Time-indexed records	Reproducible processing rule
Duplicate and validity control	4	Remove duplicate records by platform, spatial unit, and time interval. Check $v_{cur}$, $v_{ff}$, $TT_{cur}$, $TT_{ff}$, confidence, and road-closure status.	Invalid-record and audit log	Field validity from [13,14]
Road-closure handling	5	Store road-closure records in the audit table but exclude them from regular speed, travel-time, TTI, and CI calculation.	Closure-audit records	Study-defined audit rule
Cleaned dataset generation	6	Generate the cleaned dataset ${𝒟}_{c}$ using the valid-record rule in Eq. 1.	Cleaned dataset ${𝒟}_{c}$	Study-defined rule based on [13,14]
Availability assessment	7	Compute daily availability and missing-interval ratio using Eq. 2.	Availability indicators	Traffic monitoring QA/QC [1]
Traffic indicator generation	8	Compute SRR, TTI, ETT, and CI using Eq. 3.	Indicator set ℐ	Traffic performance indicators [19–21]
Temporal harmonization	9	Aggregate TomTom 15 min records into 30 min periods without upsampling HERE data using Eq. 4.	TomTom 30 min records	FCD/probe-data aggregation [2,7–9,22–25]
Spatial harmonization	10	Assign TomTom virtual points and HERE returned road segments to three common benchmark blocks using Eq. 5.	Block-level TomTom and HERE records	Network/block aggregation [2,7–9,22–25]
Matched sample construction	11	Join TomTom and HERE records by benchmark block and 30 min period. Keep only records where both platforms have valid benchmark values.	Matched sample set ${𝒟}_{m}$	Matched benchmark design
Benchmark calculation	12	Compute Pearson correlation, Spearman correlation, and peak-overlap index using Eqs. 6–8.	Benchmark results ℬ	Pearson, Spearman, and Jaccard definitions [26–28]
Sensitivity assessment	13	Compute block-specific, daily-stratified, bootstrap-confidence, threshold-sensitivity, and temporal-lag checks from the matched benchmark table.	Sensitivity outputs	Sensitivity analysis of the matched benchmark
Reproducible export	14	Export ${𝒟}_{c}$, ℐ, ${𝒟}_{m}$, ℬ, sensitivity outputs, figure-reproduction data, and data dictionary.	Reproducible output files	Reproducibility package

*Note.* ℛ denotes raw platform traffic-flow records; ${𝒟}_{c}$ denotes the cleaned dataset; ℐ denotes the traffic indicator set; ${𝒟}_{m}$ denotes the matched TomTom–HERE sample set; and ℬ denotes the benchmark results. $v_{cur}$ and $v_{ff}$ represent current speed and free-flow speed. $TT_{cur}$ and $TT_{ff}$ represent current travel time and free-flow travel time. Road-closure records are retained for audit review but are excluded from regular traffic-state indicator calculation.

Table 3 separates data-quality checks from traffic-state analysis. This separation is important because missing records, invalid values, and closure flags describe data usability, whereas speed, delay, TTI, and CI describe traffic state. Missing platform observations are not interpolated. Interpolation could make the data appear more complete than they were. The cleaning step does not smooth valid traffic variation. It only removes or flags records that are unsuitable for regular traffic-state interpretation. Variable definitions, units, data sources, aggregation levels, calculation rules, and indicator interpretation directions are provided in S2 Table.

The target of data cleaning is the cleaned record set ${𝒟}_{c}$. The valid-record rule is defined from required platform response fields and audit constraints [13,14]. A record enters ${𝒟}_{c}$ only when it satisfies the time, field, speed, travel-time, closure, and confidence constraints:


$$\begin{array}{c} {𝒟}_{c}=\left\{r_{j}\in ℛ:C_{j}^{time}C_{j}^{field}C_{j}^{speed}C_{j}^{travel}C_{j}^{closure}C_{j}^{conf}=1\right\}. \end{array}$$ (1)

where $r_{j}$ is a raw platform record; $C_{j}^{time}$ checks whether the timestamp is valid; $C_{j}^{field}$ checks whether all core fields are present; $C_{j}^{speed}$ checks whether speed values are positive; $C_{j}^{travel}$ checks whether travel-time values are positive; $C_{j}^{closure}$ excludes road-closure records from regular traffic-state calculation; and $C_{j}^{conf}$ checks whether the confidence field exists for audit review. Road-closure records are not deleted from the audit trail. They are stored separately so that abnormal operational events can be reviewed without mixing them with ordinary traffic-state estimates.

Daily availability is used as a system-operation metric before traffic-state interpretation. The completeness logic follows traffic monitoring data-quality reporting [1]. It is not a congestion metric. Let $N_{d}^{obs}$ be the number of valid TomTom records on date *d*, and let $R^{exp}$ be the expected number of records on a complete monitoring day. This study uses $R^{exp}=4032$, which equals 42 fixed virtual monitoring points multiplied by 96 daily 15 min time slots:


$$\begin{array}{c} A_{d}=\frac{N_{d}^{obs}}{R^{exp}}\times 100\%,\;MIR_{d}=100\%-A_{d},\;R^{exp}=4032. \end{array}$$ (2)

where $A_{d}$ is daily availability and $MIR_{d}$ is the missing-interval ratio. A higher $A_{d}$ indicates a more complete valid return from the acquisition system. Availability is interpreted as data completeness, not as a measure of congestion intensity.

Traffic-state indicators are generated from speed and travel-time fields returned by TomTom. Speed, travel time, travel-time index, and congestion-index measures are widely used in highway capacity analysis and congestion reporting [19–21]. Four indicators are used: speed reduction ratio, travel time index, extra travel time, and congestion index:


$$\begin{array}{cc} {SRR}_{i,t} & =\frac{v_{ff,i,t}-v_{cur,i,t}}{v_{ff,i,t}},\\ {TTI}_{i,t} & =\frac{TT_{cur,i,t}}{TT_{ff,i,t}},\\ {ETT}_{i,t} & =TT_{cur,i,t}-TT_{ff,i,t},\\ {CI}_{i,t} & =\left({TTI}_{i,t}-1\right)\times 100\%. \end{array}$$ (3)

where *i* is the virtual monitoring point and *t* is the 15 min interval. ${SRR}_{i,t}$ is dimensionless and measures relative speed loss. ${TTI}_{i,t}$ is the ratio of current travel time to free-flow travel time. ${ETT}_{i,t}$ is extra travel time in seconds. ${CI}_{i,t}$ is the congestion index in percentage form. A higher SRR, TTI, ETT, or CI indicates a worse traffic state.

The TomTom–HERE benchmark is calculated only during the overlapping benchmark period from 8 to 31 December 2025. The benchmark first harmonizes TomTom point-level records and HERE segment-level records to the same 30 min temporal scale and the same three benchmark blocks. This aggregation step follows common FCD and probe-data processing practice [2,7–9,22–25]. TomTom 15 min observations are aggregated to 30 min periods without upsampling HERE data:


$$\begin{array}{c} {\bar{x}}_{i,\tau }^{T}=\frac{1}{n_{\tau }}\sum_{t\in \tau }x_{i,t}^{T}. \end{array}$$ (4)

where ${\bar{x}}_{i,\tau }^{T}$ is the TomTom aggregated value for point *i* and 30 min period τ, $x_{i,t}^{T}$ is the raw 15 min TomTom observation, and $n_{\tau }$ is the number of valid observations within τ. HERE records are not temporally upsampled because doing so would create artificial benchmark observations.

The 42 TomTom virtual points and HERE returned road segments are assigned to the three benchmark blocks described in Table 1. Block-level aggregation is defined as follows:


$$\begin{array}{c} {\bar{x}}_{b,\tau }^{T}=\frac{1}{n_{b,\tau }}\sum_{i\in B_{b}}{\bar{x}}_{i,\tau }^{T},\;{\bar{x}}_{b,\tau }^{H}=\frac{1}{m_{b,\tau }}\sum_{s\in S_{b}}x_{s,\tau }^{H}. \end{array}$$ (5)

where $B_{b}$ is the set of TomTom points assigned to block *b*, $S_{b}$ is the set of HERE returned road segments within block *b*, $n_{b,\tau }$ is the number of valid TomTom point observations, and $m_{b,\tau }$ is the number of valid HERE segment records. This step does not treat TomTom points and HERE road segments as the same spatial units. It only creates a common block–time unit for comparison.

Matched benchmark samples are constructed by joining records by block ID and 30 min timestamp. The main benchmark variables are TomTom mean CI and HERE mean jam factor. A record enters the benchmark sample only when both platforms have valid values for the same block and period. Pearson correlation assesses the linear association between the two benchmark signals [26]:


$$\begin{array}{c} r=\frac{\sum_{k=1}^{K}\left(CI_{k}^{T}-\overset{―}{CI^{T}}\right)\left(JF_{k}^{H}-\overset{―}{JF^{H}}\right)}{\sqrt{\sum_{k=1}^{K}{\left(CI_{k}^{T}-\overset{―}{CI^{T}}\right)}^{2}}\sqrt{\sum_{k=1}^{K}{\left(JF_{k}^{H}-\overset{―}{JF^{H}}\right)}^{2}}}. \end{array}$$ (6)

where *k* is a matched block–time observation, *K* is the total number of matched observations, $CI_{k}^{T}$ is the TomTom congestion index for observation *k*, $JF_{k}^{H}$ is the HERE jam factor for observation *k*, and $\overset{―}{CI^{T}}$ and $\overset{―}{JF^{H}}$ are their sample means.

Spearman correlation assesses rank-based consistency because the two platforms do not share the same measurement scale [27]:


$$\begin{array}{c} \rho =1-\frac{6\sum_{k=1}^{K}d_{k}^{2}}{K(K^{2}-1)}. \end{array}$$ (7)

where $d_{k}$ is the rank difference for the *k*-th matched observation.

The peak-overlap index uses a Jaccard form to evaluate whether high-pressure periods identified by the two platforms overlap [28]:


$$\begin{array}{cc} S_{T} & =\left\{k:CI_{k}^{T}\geq Q_{\alpha }\left(CI^{T}\right)\right\},\\ S_{H} & =\left\{k:JF_{k}^{H}\geq Q_{\alpha }\left(JF^{H}\right)\right\},\\ PO & =\frac{\left|S_{T}\cap S_{H}\right|}{\left|S_{T}\cup S_{H}\right|}. \end{array}$$ (8)

where $Q_{\alpha }(\cdot )$ is the upper-quantile threshold fixed in the reproduction script and data dictionary, $S_{T}$ is the set of high-pressure periods identified by TomTom, and $S_{H}$ is the set of high-congestion periods identified by HERE. A higher *PO* means stronger overlap in detected high-pressure periods.

Because the matched benchmark data are repeated block–time observations, the pooled correlation is not interpreted as an independent ground-truth validation statistic. The benchmark indicators are used to describe agreement at the common block–time scale. To address the repeated-observation structure, the benchmark workflow includes five sensitivity checks. First, block-specific estimates are calculated separately for the three benchmark blocks. Second, daily-stratified correlations are calculated to check whether the pooled result is dominated by a small number of days. Third, bootstrap 95% confidence intervals are calculated by resampling matched block–time records. Fourth, the peak-overlap index is recalculated under alternative upper-quantile thresholds. Fifth, a ±30 min temporal-lag check is used to test whether the benchmark agreement depends on minor timing offsets. These checks support the comparison but do not prove absolute accuracy.

The final benchmark output includes descriptive statistics, matched observation counts, pooled consistency indicators, block-specific indicators, daily-stratified summaries, bootstrap confidence intervals, threshold-sensitivity results, and temporal-lag sensitivity results. These outputs are exported with the cleaned tables, matched benchmark table, data dictionary, and reproduction scripts so that the benchmark can be independently inspected and rerun.

## Results

**Fig 3:**
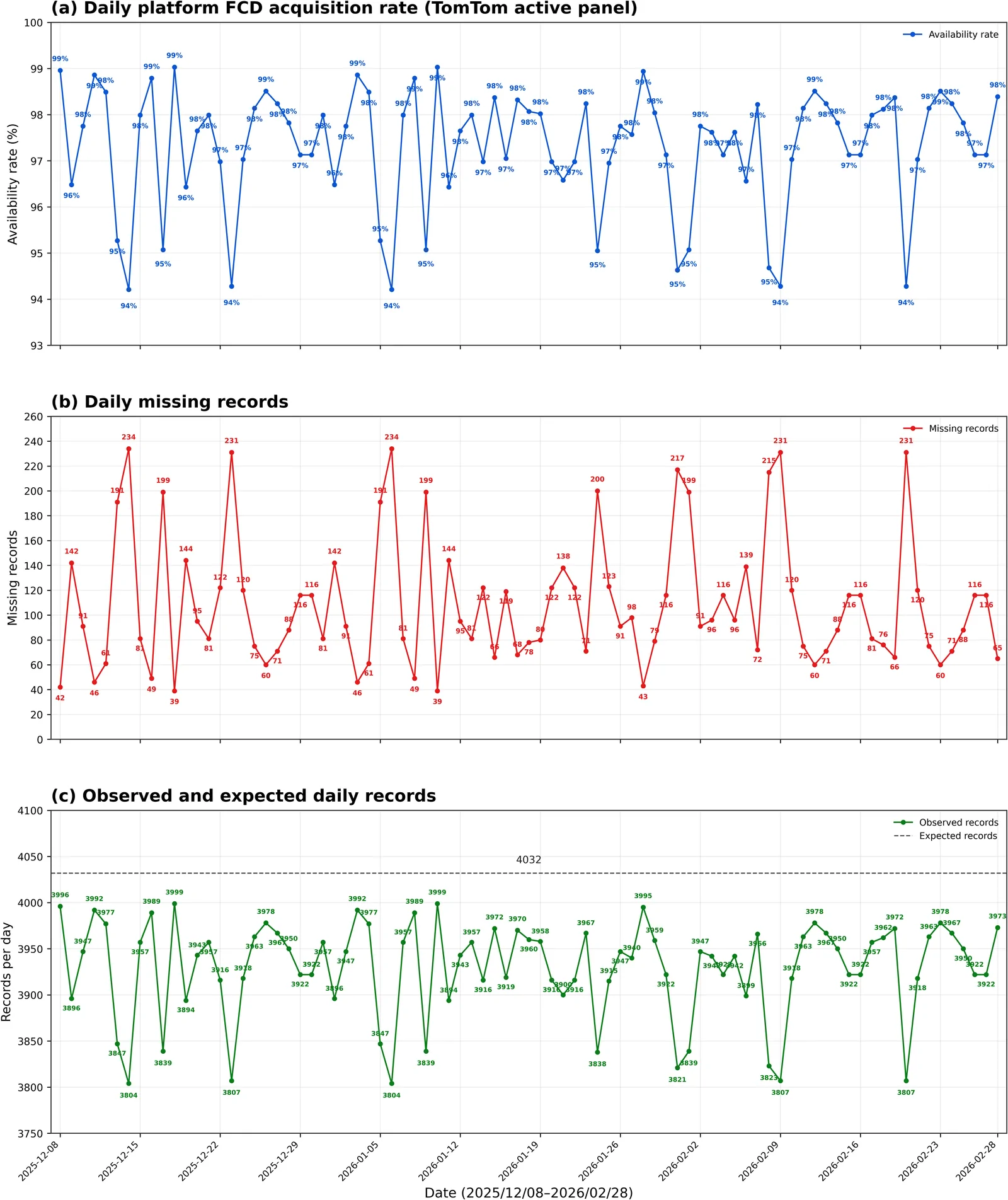
Runtime availability of the 42-point TomTom virtual monitoring panel. *Note.* The expected complete count is 4032 records per day, calculated as 42 virtual monitoring points multiplied by 96 daily 15 min intervals. Availability is interpreted as a data-completeness indicator, not as a traffic congestion indicator.

**Fig 4:**
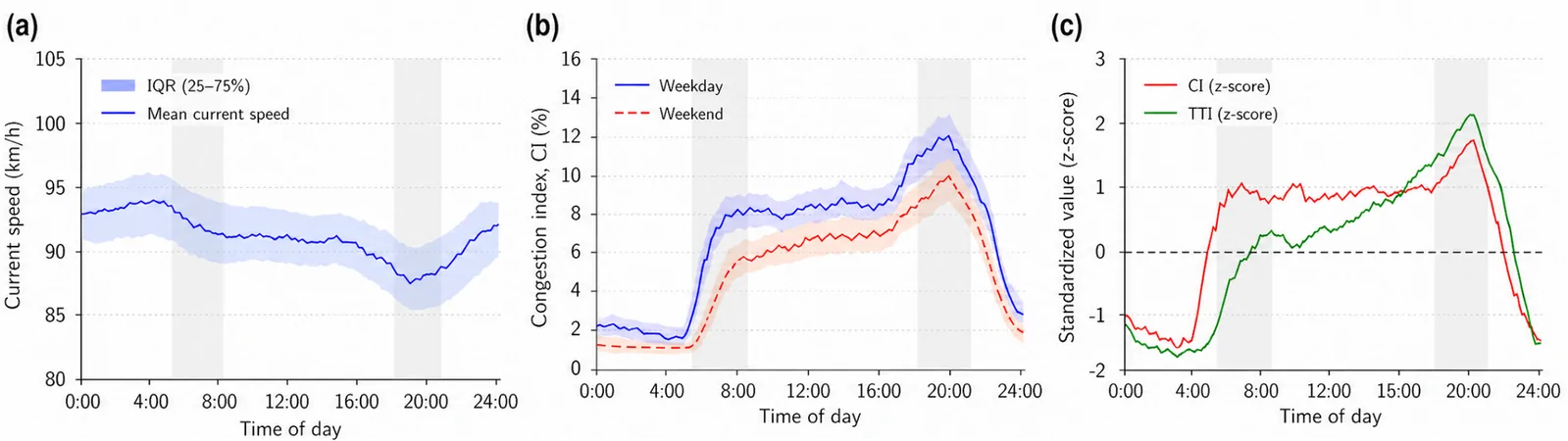
Traffic-state detection output of the TomTom virtual monitoring panel. *Note.* CI = congestion index; TTI = travel time index. The evening pressure window is interpreted as descriptive traffic-state detection evidence from platform FCD rather than as a causal explanation of congestion.

**Fig 5:**
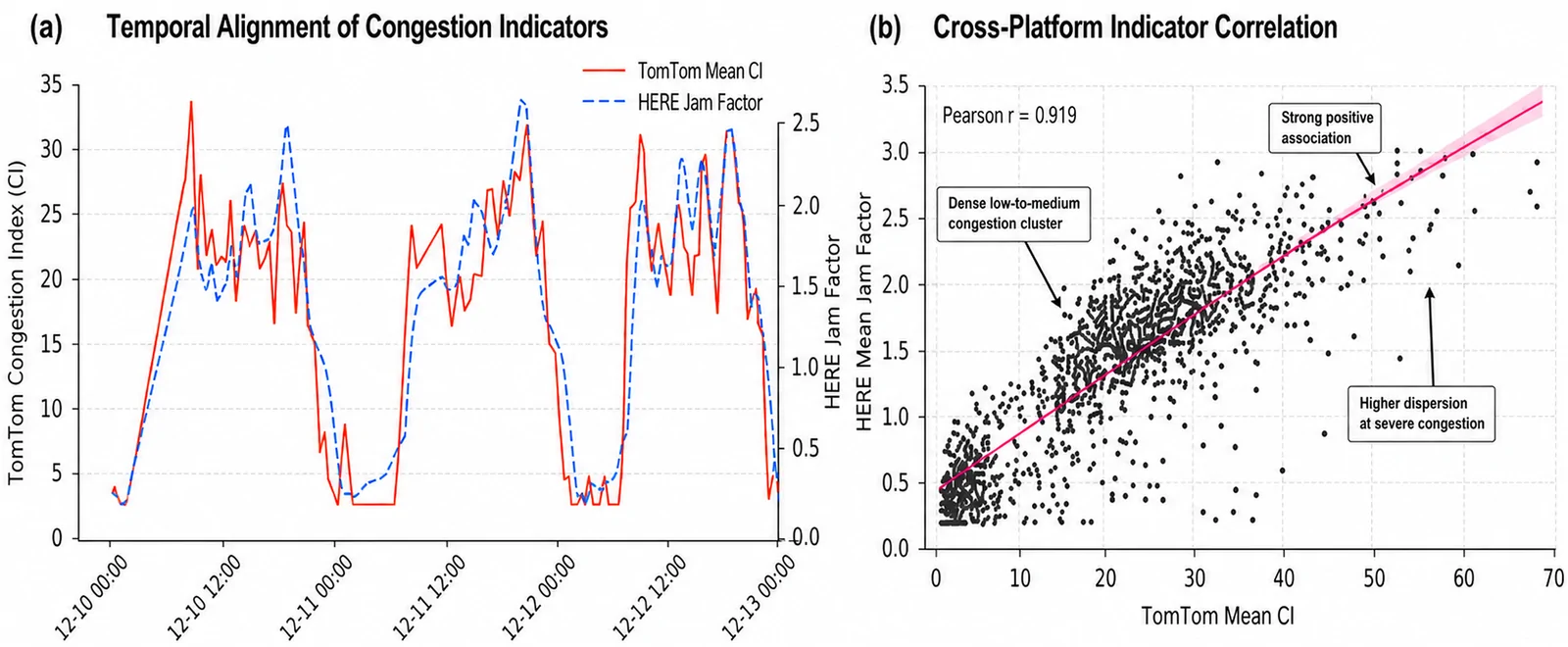
TomTom–HERE cross-platform benchmark results. *Note.* The benchmark is limited to the overlapping period from 8 to 31 December 2025. The comparison is interpreted as platform agreement after temporal and spatial harmonization, not as ground-truth validation. CI = congestion index. File name: Fig5.tiff.

The reproduction data tables underlying Figs 3–5 are provided in S1 Data.

### Runtime availability and cleaning outcomes

Before speed, travel-time, and congestion indicators are interpreted, the data stream must first be checked for completeness. Runtime availability is therefore reported as a data-completeness indicator, not as a congestion indicator. The expected complete daily count was 4032 records, calculated as 42 fixed TomTom monitoring points multiplied by 96 daily 15 min intervals. Fig 3 summarizes daily availability, daily missing records, and observed records relative to the expected complete count.

During 8 December 2025–28 February 2026, the program generated TomTom point–time records, runtime logs, cleaning logs, and daily availability statistics. The cleaning log separated missing core fields, invalid physical values, duplicate records, and road-closure-related records before the cleaned panel was used for traffic-state interpretation. Missing platform observations were not interpolated because interpolation could make the data appear more complete than they were. Road-closure records were retained in the audit trail but excluded from regular speed, travel-time, TTI, and CI calculation. Detailed data-cleaning outcomes are summarized in S3 Table.

Fig 3A shows that the daily acquisition rate remained consistently high during the monitoring period. The mean daily availability was 97.30%, indicating that the 42-point panel produced a largely complete point–time monitoring chain. This result shows that the scheduled data-collection process was stable. It should not be interpreted as evidence of absolute traffic accuracy, detector-level validity, or congestion intensity.

Fig 3B shows that missing records fluctuated by date but did not show a sustained increase or uncontrolled failure pattern. The median daily missing count was 102 records. Missing intervals may be related to network requests, platform return status, field parsing, cache access, database writing, or later processing. Because task-level logs and cleaning logs were stored together with the traffic records, abnormal days can be traced and reviewed rather than treated as unexplained data loss.

Fig 3C shows that observed daily records were generally close to the expected reference count of 4032 records per day. As summarized in Table 4, the lowest daily availability was 94.35%, corresponding to 3804 observed records, and the highest daily availability was 99.18%, corresponding to 3999 observed records. The standard deviation of daily availability was 1.01 percentage points, indicating limited day-to-day runtime fluctuation. Overall, the monitoring chain was not free from missing records, but it produced stable and reusable data for later speed, travel-time, congestion-index, and benchmark analyses.

**Table 4:** Cleaning and availability outcomes of the TomTom virtual monitoring panel.

Outcome domain	Indicator	Reported value	Sample basis or denominator	Interpretation
Daily availability	Expected complete records per day	4032	42 points × 96 daily 15 min slots	Reference denominator for daily panel completeness
Daily availability	Mean daily availability	97.30%	Valid observed records / expected records	High data completeness
Daily availability	Minimum daily availability	94.35% (3804/4032)	Lowest observed daily count	Lowest daily completeness remained above 94%
Daily availability	Maximum daily availability	99.18% (3999/4032)	Highest observed daily count	Near-complete daily panel return
Daily availability	Standard deviation	1.01 percentage points	Daily availability series	Limited day-to-day fluctuation
Missingness	Median daily missing records	102.00 records	Daily missing-count series	Missingness was generally limited
Cleaning audit	Road-closure records	Stored separately for audit review	Platform road-closure field and cleaning log	Excluded from regular traffic-state indicator calculation

*Note.* Values summarize the availability and cleaning outcomes of the TomTom virtual monitoring panel. Daily availability is calculated against 4032 expected records per day. The table reports data completeness and cleaning-audit handling; it should not be interpreted as a congestion summary.

The availability results answer the first research question by showing that a fixed-point platform FCD panel can provide stable daily acquisition coverage for the case corridor. However, availability only demonstrates that the monitoring chain returned valid records at a high rate. It does not demonstrate absolute speed accuracy, detector-level validity, or independence from provider-side processing. The subsequent traffic-state and TomTom–HERE benchmark analyses are therefore interpreted only after this data-completeness check.

### Traffic-state detection capability

After the availability and cleaning checks, the cleaned TomTom panel showed a repeated evening traffic-pressure window. This result is examined using three outputs: the intraday speed profile, the weekday–weekend CI profile, and the standardized CI–TTI profile. Fig 4 summarizes these traffic-state detection outputs.

The speed profile is used first because speed is the most direct operational variable returned by the platform. Fig 4A shows that the mean current speed was higher in the early morning and declined in the evening. The highest value occurred around 05:00 (94.23 km/h), and the lowest value occurred around 19:30 (83.31 km/h). This pattern indicates that the workflow can identify the transition from low-pressure periods to an evening pressure window after the records have passed the availability and cleaning checks.

The weekday–weekend CI profile is used to assess whether the detected pressure pattern differs by day type. Fig 4B shows that CI was lowest in the early morning (0.05%) and reached its highest value around 19:30 (12.57%). The weekday and weekend curves were broadly similar, but weekday congestion was slightly higher. Mean weekday speed was 89.01 km/h, and mean weekend speed was 89.33 km/h. Mean weekday CI was 6.18%, and mean weekend CI was 5.75%. This result suggests that the evening pressure pattern was not explained only by a weekday–weekend split. It is better described as a repeated intraday pressure pattern in the platform FCD.

The standardized CI–TTI profile is used to check whether different pressure indicators identify the same time window. A single indicator can be affected by its unit, scale, or calculation logic. Standardization allows the CI and TTI profiles to be compared on a common scale and is useful when indicators have different units [29,30]. Fig 4C shows that standardized CI and TTI followed similar temporal profiles. TTI increased from about 1.001 to 1.126. The evening period therefore showed a synchronized speed decline, CI increase, and TTI increase. This agreement across indicators supports the internal consistency of the result.

These findings answer the second research question by showing that the fixed TomTom monitoring panel can produce interpretable intraday traffic-state patterns from platform FCD. However, the result should be interpreted as descriptive detection evidence rather than causal inference. The main analysis does not include traffic volume, vehicle class, land use, trip purpose, holidays, rainfall, incidents, or independent field detectors. Therefore, the evening pressure window identifies when the platform records indicate higher traffic pressure, but it does not by itself explain why that pressure occurred.

The cleaning and audit rules also limit how this result should be interpreted. Missing observations were not interpolated, invalid values were excluded, and road-closure records were retained for audit review but excluded from regular traffic-state indicator calculation. Consequently, Fig 4 reports traffic-state patterns from the cleaned TomTom monitoring panel, platform-derived observations after quality control rather than independent field measurements. The output can be used for corridor screening, temporal pattern recognition, and field-check prioritization, but it does not show absolute speed accuracy or detector-level validity.

### TomTom–HERE cross-platform benchmark consistency

The TomTom–HERE benchmark assessed cross-platform consistency during the overlap period. A one-month HERE sample was collected from 8 to 31 December 2025 for comparison with the TomTom panel, which continued from 8 December 2025–28 February 2026. Fig 5 summarizes the temporal alignment and scatter association between TomTom mean CI and HERE mean jam factor after temporal and spatial harmonization.

The cross-platform benchmark panel was constructed at the 30 min block scale. TomTom 15 min point-level observations were first aggregated to 30 min periods. The 42 TomTom virtual monitoring points and HERE returned road segments were then assigned to the same three benchmark blocks. Matched records were retained only when both platforms had valid values for the same benchmark block and 30 min period. This harmonization step was necessary because TomTom and HERE do not return identical spatial units, update intervals, or measurement scales.

The final matched subset contained 3243 matched 30 min block–time observations. Block 1, Block 2, and Block 3 contained 1082, 1081, and 1080 observations, respectively. Because the records are repeated block–time observations, the pooled statistics are interpreted as descriptive consistency indicators.

Fig 5A shows that TomTom mean CI and HERE mean jam factor generally moved in similar directions during the main rising and falling phases. The temporal alignment was especially visible during higher-pressure periods. This pattern shows that the two platforms captured similar corridor-level pressure changes after block–time aggregation.

Fig 5B shows a positive pooled association between TomTom mean CI and HERE mean jam factor across the matched sample. After pooling all blocks, the TomTom mean CI was 5.677%, and the HERE mean jam factor was 0.358. The Pearson correlation was 0.919, the Spearman rank correlation was 0.656, and the Jaccard peak-overlap index was 0.814. The high Pearson coefficient indicates strong pooled linear co-movement between TomTom CI and HERE jam factor. The lower Spearman coefficient indicates weaker rank-order agreement across all matched block–time observations. The peak-overlap value indicates that the two platforms identified many of the same high-pressure periods.

The difference between Pearson and Spearman results is important for interpretation. The high Pearson correlation shows that the two signals rose and fell together in the pooled sample. The lower Spearman correlation shows that the rank order of individual block–time observations was less consistent. This pattern is reasonable because TomTom CI and HERE jam factor are not the same variable. They are produced by different platform pipelines, spatial definitions, aggregation rules, confidence logic, and traffic-state scaling methods.

Residual disagreement is expected because the platforms use different road-segment definitions, update intervals, confidence rules, spatial aggregation units, and vendor processing pipelines.

The sensitivity analyses are reported numerically in S4 Table. Bootstrap resampling of the released matched table produced a pooled Pearson estimate of 0.919 (95% CI: 0.915–0.933) and a pooled Spearman estimate of 0.656 (95% CI: 0.627–0.682). Across the 24 daily strata, Pearson coefficients ranged from 0.810 to 0.975 and Spearman coefficients from 0.501 to 0.762. Under temporal shifts of −30, 0, and +30 min, the pooled Pearson coefficients were 0.921, 0.919, and 0.912, while the corresponding Spearman coefficients were 0.645, 0.656, and 0.661. The pooled peak-overlap Jaccard index was 0.566, 0.715, and 0.870 at the upper 10%, 15%, and 20% thresholds, respectively. Block-specific results were heterogeneous: Block 3 showed strong agreement (Pearson *r* = 0.910; Spearman ρ=0.898), whereas Blocks 1 and 2 showed weak or negative associations (Pearson r=−0.051 and −0.040, respectively). Thus, the sensitivity checks support the stability of the pooled temporal pattern across days, thresholds, bootstrap samples, and small time offsets, while showing that the strength of agreement was not spatially uniform across the three blocks.

Overall, the TomTom–HERE benchmark answers the third research question by showing a strong pooled association after temporal and spatial harmonization, together with substantial block-level heterogeneity. Agreement was strong in Block 3 but weak in Blocks 1 and 2, indicating that the pooled result should not be generalized uniformly across the full corridor.

## Discussion

### Reliability and methodological contribution of the detection workflow

The methodological contribution lies in treating FCD monitoring as a data-production and audit process rather than only as retrospective analysis of a completed dataset. The coordinates, acquisition schedules, field parsing, validity rules, task logs, database outputs, indicator calculations, and benchmark transformations are documented and executed as one chain. Acquisition failures, missing intervals, and spatial-matching decisions are therefore measurable outputs that can be inspected before traffic indicators are interpreted. By contrast, studies that start from previously collected FCD typically evaluate the analytical value of the available records but cannot fully audit how the monitoring stream was generated.

The daily availability result shows that the data-collection process was stable before traffic-state analysis. Studies on commercial FCD reliability have often focused on penetration rate, speed accuracy, probe representativeness, and the relationship between FCD and independent observations [3–6]. These issues are important for validation, but they do not directly show whether an operational corridor-monitoring system can continuously return usable records. In this study, the TomTom panel achieved a mean daily availability of 97.30% under the 4032-records-per-day reference. This addresses a practical problem: speed, delay, TTI, and CI should be interpreted only after data completeness has been checked.

The fixed 42-point panel improves spatial repeatability compared with ad hoc data extraction. Previous studies using FCD, Bluetooth data, probe-vehicle data, and multi-source traffic records have shown that new traffic data sources can support speed estimation, volume inference, and network-wide traffic-state analysis [7–9]. Other work has used connected-vehicle trajectories or FCD streams for dynamic estimation, forecasting, and traffic-management applications [22–25]. This study builds on those results but applies a fixed-coordinate corridor panel rather than changing spatial samples. The same 42 TomTom anchors are retained across the monitoring period, and the same point-to-block structure is used for benchmark aggregation. This design reduces the spatial repeatability problem in corridors with limited detectors and limited public traffic records.

The workflow separates data completeness, traffic-state detection, and platform comparison. In conventional traffic-data analysis, these layers can be mixed: a complete dataset may be treated as a reliable traffic estimate, or a strong platform correlation may be described as validation. This study avoids that problem by treating daily availability as data completeness, TomTom indicators as descriptive traffic-state results, and TomTom–HERE agreement as platform agreement.

### Cross-platform benchmark interpretation and deployment value

The TomTom–HERE benchmark evaluates agreement between two vendor-processed signals rather than accuracy against an independent field reference. Validation-oriented FCD studies commonly use Bluetooth observations, field detectors, signalized-intersection data, or controlled probe runs [3–7], none of which were available continuously along the full Kuching–Telok Melano corridor. HERE was therefore used to test whether the TomTom-derived pressure pattern appeared in another platform after temporal and spatial harmonization.

The pooled benchmark result shows strong agreement at the common block–time scale. The released matched table yielded a pooled Pearson coefficient of 0.919 and a Spearman coefficient of 0.656, with bootstrap 95% confidence intervals of 0.915–0.933 and 0.627–0.682, respectively. However, the block-specific analysis showed that the pooled result was not spatially uniform: Block 3 showed strong agreement, while Blocks 1 and 2 showed weak associations. This distinction is important because TomTom CI and HERE jam factor are different variables generated through different platform pipelines, spatial definitions, confidence rules, and aggregation procedures.

The one-month HERE sample tests the benchmark procedure but should not be read as long-term validation. A one-month HERE sample was collected from 8 to 31 December 2025 for comparison with the TomTom panel, which continued from 8 December 2025–28 February 2026. This design is sufficient to test 30 min temporal matching, three-block spatial aggregation, matched-sample construction, and consistency calculation. However, it cannot show whether the TomTom–HERE relationship remains stable across longer seasonal periods, holidays, rainfall events, incidents, or abnormal disruptions.

The observed agreement may still reflect shared road-network geometry, overlapping probe influences, and common traffic demand, while differences can arise from vendor-specific map matching, smoothing, confidence rules, and aggregation.

The workflow can provide a low-cost screening step before agencies invest in permanent sensors. Fixed counters, Bluetooth readers, cameras, and other roadside sensors can provide stronger field evidence, but they require installation, power supply, communication infrastructure, maintenance, and institutional resources [1]. These requirements are difficult to satisfy continuously along long data-scarce corridors. The proposed workflow does not replace calibrated field detectors. Instead, it helps agencies identify high-pressure time windows, sections requiring field checking, and candidate locations for future permanent sensors.

The same structure can be applied to other data-scarce corridors. New virtual points can be added by updating the coordinate list, new benchmark blocks can be added by updating the spatial allocation table, and longer monitoring can be implemented by extending scheduled acquisition tasks. Other data streams, such as rainfall records, crash data, traffic counts, camera-based observations, or additional platform services, can be connected when they include time and spatial identifiers. This extends previous FCD applications because the monitoring design can be updated without rebuilding the whole analysis.

The Pan Borneo Highway case shows why this workflow is relevant for regional corridor management. The Kuching–Telok Melano section combines urban-fringe movement, intermediate town-node traffic, coastal-settlement access, tourism, freight, and regional travel functions. These mixed functions create repeated monitoring needs, but permanent detector coverage and public traffic-state records are limited. The proposed workflow provides an initial monitoring layer for such conditions. It can support screening, temporal pattern recognition, field-validation prioritization, and longer-term monitoring design before agencies commit to more expensive sensing infrastructure.

### Limitations and future research

The main limitation is the absence of an independent ground-truth detector. The TomTom–HERE benchmark shows external consistency between two platform signals, but it cannot quantify true speed error, true travel-time error, or detector-level accuracy. This affects how the speed, delay, TTI, and CI results should be read. Future work should add roadside detectors, controlled probe runs, Bluetooth observations, camera-based measurements, or temporary field campaigns to quantify absolute errors.

Previous FCD validation studies show that probe-data speed accuracy depends on context. Speed differences between FCD and field reference measurements may be small on well-covered road sections but can increase when probe penetration is low, traffic states change rapidly, road geometry is complex, or vendor aggregation units do not match the field reference location [3–7]. Because this study lacks detector-based ground truth, the TomTom speed, delay, TTI, and CI values should be read as platform-derived indicators, not as absolute field measurements. Differences between TomTom and HERE may also arise from different probe populations, map-matching rules, road-segment definitions, update intervals, confidence filtering, smoothing procedures, and spatial aggregation methods.

The cross-platform benchmark is limited by the one-month HERE sample. This overlap window is useful for testing the benchmark procedure during a matched period. However, it cannot evaluate whether the same consistency remains stable across longer monitoring periods, rainfall seasons, holiday periods, incidents, or major demand changes. Future research should extend HERE sampling or add other platform sources across multiple months to evaluate temporal stability.

The traffic-state results remain descriptive because explanatory variables were not included. The evening pressure window is internally consistent across speed, CI, and TTI, but the analysis cannot determine whether it was caused by commuting, freight activity, tourism travel, rainfall, incidents, road geometry, access control, or local land-use conditions. Future work should add rainfall data, incident records, land use, traffic volume, vehicle class, and road geometry to support causal analysis.

The workflow also needs to be tested on other corridors and road types. This study provides methodological evidence from one data-scarce highway corridor in Sarawak. Other corridors may have different probe penetration, platform coverage, road geometry, traffic composition, and vendor response stability. Future studies should apply the same fixed-panel, audit-based, and block-harmonized workflow to other Pan Borneo Highway sections and other regional corridors. This would show which parts can be reused and which parts need local calibration.

## Conclusion

This study shows that a script-based FCD workflow can support traffic monitoring on a data-scarce highway corridor. The workflow combines scheduled data collection, rule-based quality control, database storage, traffic indicator calculation, and cross-platform comparison. The empirical case used 42 fixed TomTom virtual monitoring points on the Kuching–Telok Melano section of the Pan Borneo Highway. A one-month HERE sample from 8 to 31 December 2025 was used as an external benchmark module to test cross-platform consistency during a clearly overlapping period with the longer TomTom monitoring panel.

The empirical results show that the TomTom monitoring panel produced stable outputs. Using 4032 expected records per day as the reference, the mean daily availability was 97.30%, indicating high data completeness. The cleaned traffic indicators identified a repeated evening pressure window around 19:30, with synchronized speed decline, CI increase, and TTI increase. The TomTom–HERE benchmark showed strong pooled consistency across 3243 matched block–time observations, and the completed sensitivity analyses remained stable across daily strata, bootstrap samples, alternative peak thresholds, and ±30 min time offsets. The block-specific analysis nevertheless showed substantial spatial heterogeneity, with strong agreement in Block 3 and weak associations in Blocks 1 and 2. These results indicate that platform FCD can support repeatable corridor screening when data collection, cleaning, storage, indicator calculation, and comparison are managed together.

The proposed workflow should be used as a supplementary screening method rather than as a replacement for calibrated field detectors. It can support identification of high-pressure periods, block-level diagnosis, field-check prioritization, and longer-term monitoring design where permanent sensors are limited. Future work should add independent field measurements, extend the cross-platform overlap beyond the initial one-month HERE period, incorporate explanatory variables such as rainfall, incidents, traffic volume, and road geometry, and test the workflow on additional data-scarce corridors.

## Supporting information

S1 Data Reproduction data tables for Figs 3–5. This file includes the availability data for Fig 3, the traffic-state detection profile for Fig 4, and the TomTom–HERE benchmark table for 8–31 December 2025. The archived reproducibility package is available on Zenodo: https://doi.org/10.5281/zenodo.21093286.
(XLSX)

S1 Table TomTom virtual monitoring point and benchmark-block configuration. This table reports the TomTom virtual monitoring point ID, coordinate, corridor order, assigned HERE benchmark block, and key spatial notes for the fixed-point monitoring panel and benchmark-block design.
(XLSX)

S2 Table Data dictionary and indicator direction. This table defines each variable, unit, data source, aggregation level, calculation rule, and interpretation direction used in the workflow and benchmark analysis.
(XLSX)

S3 Table Cleaning outcome summary. This table reports raw records, valid records, missing-core-field records, non-positive speed records, non-positive travel-time records, closure-related records, duplicate records, and cleaning pass rates.
(XLSX)

S4 Table TomTom–HERE benchmark sensitivity results. This table reports block-specific consistency, daily-stratified consistency, bootstrap confidence intervals, upper-quantile threshold sensitivity, and temporal-lag sensitivity generated from the released matched benchmark table.
(XLSX)

S1 Text Computing environment and implementation details. The acquisition pipeline ran on a 64-bit Windows x64 workstation with an Intel Core i7-10700 processor at 2.90 GHz, 16.0 GB RAM, 238 GB SSD storage, and 1.82 TB HDD storage. Java 17.0.18 and Spring Boot 3.3.4 managed scheduled API acquisition. PostgreSQL 17 stored cleaned traffic records, acquisition status, abnormal-record logs, and audit information [18]. Redis 3.2.100 cached monitoring-point configurations, platform access-key pools, user-agent lists, and service heartbeat status [31]. Python 3.10 with pandas and NumPy supported data cleaning, aggregation, indicator calculation, sensitivity analysis, and figure reproduction [15–17]. The available graphics devices were an NVIDIA GeForce RTX 2060 SUPER and Intel UHD Graphics 630; neither was used as a primary computing component. Each scheduled task recorded its start time, request time, platform return status, valid-record count, abnormal-record count, database-write status, and error message. This information is reported for implementation reproducibility rather than as a computing-performance benchmark.
(DOCX)
