# Supplementary material for: Reproducible floating car data monitoring for data-scarce highway corridors: A TomTom–HERE benchmark study
Source: PLoS One. 2026 Aug 31;21(8):e0357158. doi: 10.1371/journal.pone.0357158 (PMC13529016; doi:10.1371/journal.pone.0357158)
Supplement: Supplementary file 1 [file pone.0357158.s006.docx]

**S1 Text. Computing environment and implementation details**

**Computing environment**

The acquisition pipeline ran on a 64-bit Windows x64 workstation with an Intel Core i7-10700 processor at 2.90 GHz, 16.0 GB RAM, 238 GB SSD storage, and 1.82 TB HDD storage.

**Acquisition implementation**

Java 17.0.18 and Spring Boot 3.3.4 managed scheduled API acquisition.

**Data storage and caching**

PostgreSQL 17 stored cleaned traffic records, acquisition status, abnormal-record logs, and audit information [15]. Redis 3.2.100 cached monitoring-point configurations, platform access-key pools, user-agent lists, and service heartbeat status [16].

**Analysis and figure reproduction**

Python 3.10 with pandas and NumPy supported data cleaning, aggregation, indicator calculation, sensitivity analysis, and figure reproduction [12--14]. The available graphics devices were an NVIDIA GeForce RTX 2060 SUPER and Intel UHD Graphics 630; neither was used as a primary computing component.

**Task-level audit logging**

Each scheduled task recorded its start time, request time, platform return status, valid-record count, abnormal-record count, database-write status, and error message. This information is reported for implementation reproducibility rather than as a computing-performance benchmark.
