# Supplementary material for: Reproducible floating car data monitoring for data-scarce highway corridors: A TomTom–HERE benchmark study
Source: PLoS One. 2026 Aug 31;21(8):e0357158. doi: 10.1371/journal.pone.0357158 (PMC13529016; doi:10.1371/journal.pone.0357158)
Supplement: Supplementary file 2 [file pone.0357158.s008.docx]

**Response to the Editor and Reviewers**

*Manuscript title: Reproducible floating car data monitoring for data-scarce highway corridors: A TomTom--HERE benchmark study*

Dear Editor and Reviewers,

We sincerely thank the Editor and the reviewers for their careful reading and constructive comments. The comments helped us improve the manuscript substantially. We revised the manuscript to clarify the data sources, the benchmark design, the figure-source information, the evidence boundary of the TomTom--HERE comparison, and the reproducibility package. We have prepared a clean revised manuscript and a highlighted revised manuscript. We also provide this point-by-point response. We describe each change below. We quote the revised manuscript text where useful.

Responses to the Editor

**Editor comment 1: Please provide a response letter, a revised manuscript with changes marked, and a clean revised manuscript.**

Response: Thank you for this instruction. We have prepared all requested files. We have prepared a clean revised manuscript, a highlighted revised manuscript, and this detailed response letter.

**Editor comment 2: Please provide more information about the data sources and confirm compliance with the terms and conditions of the data sources.**

Response: Thank you for pointing this out. We revised the Methods section and the Data Availability Statement to provide more detailed information about the data sources and redistribution conditions. We reviewed the relevant TomTom and HERE API documentation and data-use terms before preparing the public reproducibility package. Based on this review, we designed the public release to include only cleaned, derived, aggregated, and de-identified materials that are needed for academic reproducibility. We now describe the TomTom and HERE data sources, the sampling periods, the temporal resolutions, the spatial units, the main variables, and the harmonization rules. We also state that authorized developer/API access was used for data collection. To respect the platform terms and protect restricted materials, we do not redistribute API keys, account credentials, access tokens, private request headers, full key-bearing request URLs, complete raw API dumps, full vendor-returned road geometries, local database dumps, or private source-data folders. Instead, the public GitHub repository and Zenodo archive provide cleaned tables, derived benchmark outputs, scripts, spatial metadata, API field-mapping notes, and data dictionaries. These materials are de-identified and prepared as a reproducibility package for academic review and reuse. Readers can use them to reproduce the reported figures, tables, and benchmark results without accessing restricted platform materials.

Change made: We added Table 2 and added a paragraph on data-source terms and redistribution restrictions in the Methods section. We also revised the Data Availability Statement to describe the Zenodo archive, GitHub repository, scripts, spatial metadata, API documentation, and excluded restricted materials.

Revised manuscript text: The collection and analysis procedures were designed to comply with the applicable terms and conditions of the TomTom and HERE data sources at the time of data collection. The study used authorized developer/API access to obtain platform traffic-flow records for academic corridor-monitoring analysis. API keys, account credentials, access tokens, private request headers, full key-bearing request URLs, complete raw API dumps, full vendor-returned road geometries, local database dumps, and private source-data folders are not redistributed. (revised manuscript, lines 175--185)

Revised manuscript text: The cleaned data tables, processing scripts, benchmark scripts, configuration files, figure data, spatial metadata, API documentation, and data dictionary are archived on Zenodo with DOI: https://doi.org/10.5281/zenodo.21093286. (revised manuscript, lines 676--679)

**Editor comment 3: Please address the possible copyright issue in the map figures and use a suitable public-domain or compatible basemap.**

Response: Thank you for raising this important issue. We revised the figure-source statement and changed the figure design to remove possible map-copyright concerns. In the revised manuscript, Fig 1 uses public-domain Natural Earth vector layers as the basemap. We also state clearly that no Google Maps, Google Earth, proprietary satellite imagery, or proprietary web-map tiles were used. In addition, we revised the previous map-based Fig 3. The revised Fig 3 is now a runtime availability plot, not a map figure. It does not contain any basemap, satellite image, or proprietary web-map tile. We also added Natural Earth as a reference to document the public-domain source.

Change made: We revised the Fig 1 note, replaced the previous map-based Fig 3 with a runtime availability plot, and added Reference 29 for the Natural Earth terms of use.

Revised manuscript text: Panels A and B were drawn using public-domain Natural Earth basemap vector layers [29]. The corridor line, monitoring points, benchmark-block boundaries, labels, and panel C schematic were generated by the authors from the study coordinate configuration and analytical design. No Google Maps, Google Earth, proprietary satellite imagery, or proprietary web-map tiles were used. (revised manuscript, lines 83--89)

Revised manuscript text: Natural Earth. Terms of use: public-domain map data [Internet]. Natural Earth; 2026 [cited 2026 May 3]. Available from: https://www.naturalearthdata.com/about/terms-of-use/ (revised manuscript, lines 791--793)

**Editor comment 4: Please follow PLOS style for figures and figure captions.**

Response: Thank you. We revised the figure structure and captions. The manuscript now uses five figures. Figure captions are placed in the manuscript after the first citation. The figures are uploaded separately as Fig1.tiff to Fig5.tiff. We also use "Fig" in the manuscript.

Change made: We revised the figure captions and file names. We also changed the supporting information from Figs 4--6 to Figs 3--5.

Revised manuscript text: S1 Data. Reproduction data tables for Figs 3--5. This file includes the availability data for Fig 3, the traffic-state detection profile for Fig 4, and the TomTom--HERE benchmark table for 8--31 December 2025. (revised manuscript, lines 794--798)

**Responses to Reviewer #1**

**Reviewer #1 comment 1: The manuscript should state more clearly that there is no independent ground-truth validation.**

Response: Thank you for this important comment. We agree. We revised the Abstract, Methods, Results, Discussion, Limitations, and Conclusion to make this point clear. We now state that the TomTom--HERE comparison is a platform comparison. It is not a validation against true field speed. We also state that no independent detector, controlled probe run, Bluetooth reader, or camera-based field measurement was available.

Change made: We added explicit wording in the Abstract and Results. We also strengthened the Limitations and Conclusion.

Revised manuscript text: These findings suggest that platform FCD can support corridor monitoring where fixed detectors are limited, but they should not be read as ground-truth validation. (revised manuscript, Abstract, page 1)

Revised manuscript text: The TomTom--HERE benchmark showed strong agreement during the overlap period, but it should not be treated as ground-truth validation. (revised manuscript, lines 434--436)

Revised manuscript text: The main limitation is the absence of an independent ground-truth detector. The TomTom--HERE benchmark shows external consistency between two platform signals, but it cannot quantify true speed error, true travel-time error, or detector-level accuracy. (revised manuscript, lines 610--613)

**Reviewer #1 comment 2: Please discuss general experience from probe-data/FCD validation studies and the possible size or nature of speed differences.**

Response: Thank you. We agree that this discussion was too limited in the earlier manuscript. We added text explaining that FCD speed accuracy is context-dependent. We did not report one fixed expected speed difference because this study does not have detector-based ground truth. Instead, we explain the conditions under which FCD speed differences can increase. These include low probe penetration, rapidly changing traffic states, complex road geometry, and mismatch between vendor aggregation units and field reference locations.

Change made: We added a new paragraph in Limitations and future research. We also strengthened the Introduction to describe the main reliability factors for commercial FCD.

Revised manuscript text: Their reliability depends on penetration rate, spatial coverage, temporal coverage, map matching, provider-specific aggregation rules, and the stability of platform responses [3--6]. (revised manuscript, lines 24--26)

Revised manuscript text: Previous FCD validation studies show that probe-data speed accuracy depends on context. Speed differences between FCD and field reference measurements may be small on well-covered road sections but can increase when probe penetration is low, traffic states change rapidly, road geometry is complex, or vendor aggregation units do not match the field reference location [3--7]. (revised manuscript, lines 617--621)

**Reviewer #1 comment 3: Please explain possible differences between TomTom and HERE and why they may occur.**

Response: Thank you. We revised the manuscript to explain that TomTom and HERE are separate platform services, but they should not be treated as fully independent ground-truth sources. We now describe possible reasons for differences between them. These include probe populations, map matching, road-segment definitions, update intervals, confidence rules, smoothing, and spatial aggregation.

Change made: We added this explanation in the Introduction, Results, Discussion, and Limitations.

Revised manuscript text: TomTom and HERE may differ because of their probe populations, map-matching logic, road-segment definitions, update intervals, confidence rules, and vendor-side aggregation procedures. They may also share overlapping data influences and therefore should not be treated as fully independent ground-truth sources. (revised manuscript, lines 39--42)

Revised manuscript text: Disagreement may arise from different road-segment definitions, different update intervals, different confidence rules, different spatial aggregation units, and different sensitivity to sparse observations. (revised manuscript, lines 484--487)

Revised manuscript text: Differences between TomTom and HERE may also arise from different probe populations, map-matching rules, road-segment definitions, update intervals, confidence filtering, smoothing procedures, and spatial aggregation methods. (revised manuscript, lines 622--626)

**Reviewer #1 comment 4: Please avoid overinterpreting the traffic-state results as causal findings.**

Response: Thank you. We agree. The revised manuscript now states that the evening pressure window is a descriptive platform-FCD pattern. We do not claim that the result explains the cause of congestion. We also state which explanatory variables were not included.

Change made: We revised the Results and Limitations to define the interpretation boundary.

Revised manuscript text: The evening pressure window is interpreted as descriptive traffic-state detection evidence from platform FCD rather than as a causal explanation of congestion. (revised manuscript, lines 390--393)

Revised manuscript text: The main analysis does not include traffic volume, vehicle class, land use, trip purpose, holidays, rainfall, incidents, or independent field detectors. Therefore, the evening pressure window identifies when the platform records indicate higher traffic pressure, but it does not by itself explain why that pressure occurred. (revised manuscript, lines 419--424)

**Responses to Reviewer #2**

**Reviewer #2 comment 1: Please clarify the novelty of the work compared with existing FCD studies and frameworks.**

Response: Thank you. We revised the Introduction and Discussion to make the novelty clearer. The revised manuscript explains that many FCD studies mainly analyze collected records. Our study focuses on a repeatable corridor monitoring workflow. It combines fixed-coordinate sampling, daily availability reporting, cleaning logs, database storage, indicator calculation, TomTom--HERE comparison, and reproducible export.

Change made: We added a horizontal comparison with previous FCD studies in the Introduction and Discussion. The revised text explains that previous studies mainly used FCD, probe-vehicle data, Bluetooth data, and stationary detectors to estimate speeds, travel times, traffic volumes, or traffic states. In comparison, our study focuses on a repeatable corridor-monitoring workflow that combines fixed virtual monitoring points, scheduled data collection, data-quality checks, block-level TomTom--HERE comparison, and clear separation between platform agreement and ground-truth validation. This revision clarifies that our study addresses the lack of a reproducible and reviewable FCD workflow for data-scarce highway corridors.

Revised manuscript text: Compared with FCD studies that mainly analyze a collected dataset, this study makes four contributions. First, it uses a fixed-coordinate monitoring panel, so repeated platform requests are made at the same corridor locations. Second, it treats daily data availability and missing-interval diagnostics as system-operation outputs before interpreting traffic-state indicators. Third, it combines acquisition, filtering, storage, audit logs, indicator generation, and benchmark calculation in one reproducible workflow. Fourth, it compares TomTom and HERE at a common block--time scale and separates platform agreement from ground-truth validation. (revised manuscript, lines 58--65)

Revised manuscript text: The main contribution of this study is a repeatable FCD workflow for corridor monitoring. (revised manuscript, lines 510--511)

**Reviewer #2 comment 2: Please define data-scarce highway corridors more clearly.**

Response: Thank you. We added a formal definition in the Introduction. We also clarified that data-scarce does not mean that no data exist. It means that available data are not sufficient for continuous, corridor-wide, and repeatable monitoring.

Change made: We added the definition at the start of the Introduction.

Revised manuscript text: In this study, a data-scarce highway corridor is defined as a long-distance road corridor where continuous fixed-detector coverage, publicly available traffic counts, calibrated travel-time surveys, and open traffic-state records are limited or absent along substantial parts of the route. This does not mean that no traffic information is available. It means that the available information is not sufficient for continuous, corridor-wide, and repeatable traffic-state monitoring. (revised manuscript, lines 6--12)

**Reviewer #2 comment 3: Please explain how the 42 TomTom virtual monitoring points were selected.**

Response: Thank you. We agree that this point needed a clearer explanation. We added a paragraph describing the point-selection logic. The 42 points were not random samples. They were fixed coordinate anchors along the main corridor. They were placed to provide repeatable spatial coverage and to represent the Kuching urban fringe, intermediate town-node sections, and western coastal-access sections. We also clarified that coordinates that could be matched by the platform to nearby non-corridor roads, minor access roads, or complex junctions were avoided. This was done to reduce traffic-data interference and to improve the distinctiveness of each acquisition point, so that repeated requests were more likely to represent the intended corridor sections without unnecessary overlap. In addition, the public GitHub repository now includes the exact latitude--longitude anchor information, the TomTom point-to-block assignment table, and the HERE benchmark-block coordinate definitions. These files allow readers to inspect how each acquisition point was located and how the points were linked to the benchmark blocks.

Change made: We added a new paragraph in the Methods section after Fig 1.

Revised manuscript text: The 42 TomTom virtual monitoring points were selected to provide repeatable spatial coverage along the approximately 130 km corridor. The points were placed on or near the main highway alignment, ordered according to corridor sequence, and distributed to represent the Kuching urban-fringe section, intermediate town-node sections, and western coastal-access sections. (revised manuscript, lines 90--94)

Revised manuscript text: The purpose of the 42-point design was not random spatial sampling, but stable repeated monitoring using fixed coordinate anchors throughout the study period. (revised manuscript, lines 95--98)

**Reviewer #2 comment 4: Please explain the three HERE benchmark blocks and the point-to-block assignment.**

Response: Thank you for pointing this out. We revised the Methods section and added Table 1 to make the spatial matching process clearer. Table 1 reports the three HERE benchmark blocks, the TomTom point IDs assigned to each block, the number of TomTom points in each block, the spatial basis, and the purpose of the block design. Each TomTom monitoring point is mapped to its corresponding HERE benchmark block in this table. We also clarified that TomTom points and HERE road segments were not treated as identical spatial units. Both sources were aggregated to common block--time records before benchmark calculation. The detailed latitude--longitude information for each TomTom point, the TomTom point-to-block assignment table, and the HERE benchmark-block coordinate definitions are provided in the public GitHub repository. These materials allow readers to check the exact spatial anchors and reproduce the block-matching process.

Change made: We added Table 1 and explanatory text before and after it.

Revised manuscript text: The HERE benchmark design used three larger corridor blocks rather than point-level matching. This was necessary because TomTom and HERE do not return identical spatial units. (revised manuscript, lines 107--109)

Revised manuscript text: Instead, both sources were harmonized to common block--time records before consistency analysis. The point-to-block assignment used for this harmonization is summarized in Table 1. (revised manuscript, lines 112--115)

Revised manuscript text: TomTom points and HERE returned road segments were not treated as identical spatial units; both were aggregated to common block--time records before benchmark calculation. Full point coordinates, HERE bounding-box coordinates, and point-to-block metadata are provided in the reproducibility package and supporting metadata. (revised manuscript, Table 1 note, page 4)

**Reviewer #2 comment 5: Please explain why the HERE benchmark period is shorter than the TomTom monitoring period.**

Response: Thank you. We revised the wording to avoid presenting the shorter HERE period as full validation. We now explain that the one-month HERE sample was used as an experimental benchmark set for the external benchmark module. It was collected during the clearly overlapping period from 8 to 31 December 2025 and was used to test the block-level TomTom--HERE matching and comparison procedure. The TomTom monitoring panel continued to 28 February 2026. We also state in the Discussion and Conclusion that this one-month HERE sample should not be read as long-term validation. In future work, we will continue HERE data collection and conduct longer-period benchmark experiments with deeper validation analysis when more overlapping data are available.

Change made: We revised the Abstract, Methods, Results, Discussion, and Conclusion.

Revised manuscript text: HERE traffic records were used as a 30 min external benchmark during the overlapping period from 8 to 31 December 2025 after temporal and spatial harmonization. (revised manuscript, Abstract, page 1)

Revised manuscript text: A one-month HERE sample was collected from 8 to 31 December 2025 for comparison with the TomTom panel, which continued from 8 December 2025 to 28 February 2026. (revised manuscript, lines 434--437)

Revised manuscript text: The one-month HERE sample tests the benchmark procedure but should not be read as long-term validation. (revised manuscript, lines 568--569)

**Reviewer #2 comment 6: Please clarify that the TomTom--HERE comparison uses repeated block--time observations and should not be interpreted as independent ground-truth validation.**

Response: Thank you. We agree that the TomTom--HERE benchmark should be interpreted as repeated block--time platform agreement, not as independent ground-truth validation. We revised the Methods, Results, Discussion, and Conclusion to make this point clear.

1. We clarified the data structure. The matched TomTom--HERE records are repeated block--time observations. They are not independent field measurements. Each record represents one benchmark block and one 30 min period after temporal and spatial harmonization.

2. We clarified the purpose of the pooled correlations. The Pearson and Spearman correlations are used to describe platform agreement at the common block--time scale. They are not used as independent ground-truth validation statistics. We now state that the pooled benchmark results should be interpreted as descriptive consistency indicators rather than as error estimates against true traffic speed.

3. We added robustness checks to reduce overinterpretation of one pooled coefficient. The revised Methods now include block-specific estimates, daily-stratified correlations, bootstrap 95% confidence intervals, upper-quantile threshold sensitivity, and a ±30 min temporal-lag check. These checks examine whether the pooled result changes with block aggregation, day-level concentration, threshold choice, or small timing offsets.

4. We revised the interpretation throughout the manuscript. We now state that the TomTom--HERE comparison supports platform agreement, but it does not establish absolute traffic-state accuracy. We also state that TomTom and HERE are vendor-processed platform signals and are not independent ground-truth sources. This wording was added to avoid overstating the benchmark result.

Change made: We added clarification in the Methods, Results, Discussion, and Conclusion. We clarified that the matched TomTom--HERE records are repeated block--time observations, that pooled correlations are descriptive platform-agreement statistics, and that the comparison should not be interpreted as independent ground-truth validation.

Revised manuscript text: Because the matched benchmark data are repeated block--time observations, the pooled correlation is not interpreted as an independent ground-truth validation statistic. The benchmark indicators are used to describe agreement at the common block--time scale. (revised manuscript, lines 320--323)

Revised manuscript text: These repeated block--time records are useful for platform comparison, but they are not independent ground-truth observations. Therefore, the pooled benchmark statistics are interpreted as descriptive consistency indicators rather than as an error estimate against true traffic speed. (revised manuscript, lines 452--457)

Revised manuscript text: The TomTom--HERE comparison supported platform agreement, but the pooled coefficients should not be treated as independent ground-truth validation statistics. (revised manuscript, lines 498--500)

**Reviewer #2 comment 7: Please report or at least locate the sensitivity checks more clearly.**

Response: Thank you. We agree that the sensitivity checks needed to be easier to find. We have now located them clearly in the Methods, Results, and Supporting Information, and we have added S5 Table to report the detailed sensitivity-check outputs.

1. In the Methods section, we now list the five sensitivity checks used in the benchmark workflow. These include block-specific estimates, daily-stratified summaries, bootstrap 95% confidence intervals, upper-quantile threshold sensitivity, and plus or minus 30 min temporal-lag sensitivity.

2. In the Results section, we now explain where the detailed sensitivity outputs are reported. We state that the block-specific estimates, daily-stratified summaries, bootstrap 95% confidence intervals, threshold-sensitivity results, and temporal-lag sensitivity results are reported in S5 Table.

3. In the Supporting Information section, we added S5 Table, titled "TomTom--HERE benchmark sensitivity results." This table reports the detailed sensitivity results generated from the released matched benchmark table.

4. We also clarified the role of these checks. They are used as robustness evidence for the TomTom--HERE platform-agreement result. They are not presented as ground-truth validation or as proof of detector-level accuracy.

Change made: We added the sensitivity-check description in the Methods section, added the location of the sensitivity outputs in the Results section, and added S5 Table in the Supporting Information.

Revised manuscript text: "To address the repeated-observation structure, the benchmark workflow includes five sensitivity checks. First, block-specific estimates are calculated separately for the three benchmark blocks. Second, daily-stratified correlations are calculated to check whether the pooled result is dominated by a small number of days. Third, bootstrap 95% confidence intervals are calculated by resampling matched block--time records. Fourth, the peak-overlap index is recalculated under alternative upper-quantile thresholds. Fifth, a plus or minus 30 min temporal-lag check is used to test whether the benchmark agreement depends on minor timing offsets." (revised manuscript, lines 323--330)

Revised manuscript text: "The detailed block-specific estimates, daily-stratified summaries, bootstrap 95% confidence intervals, upper-quantile threshold sensitivity, and plus or minus 30 min temporal-lag sensitivity results are reported in S5 Table and are reproducible from the released matched benchmark table." (revised manuscript, lines 494--497)

Revised manuscript text: "S5 Table. TomTom--HERE benchmark sensitivity results. This table reports block-specific consistency, daily-stratified consistency, bootstrap confidence intervals, upper-quantile threshold sensitivity, and temporal-lag sensitivity generated from the released matched benchmark table." (revised manuscript, lines 809--812)

**Reviewer #2 comment 8: Please clarify how road-closure records and missing observations were handled.**

Response: Thank you. We agree that this point needed a clearer explanation. We revised the Methods and Results to clarify both road-closure handling and missing-observation handling.

1. For road-closure records, we clarified that the platform JSON response includes a road-status or road-closure field. This field was parsed during data cleaning. It allows the workflow to identify whether a returned road segment was reported as closed or not passable by the platform. These records were not mixed with ordinary traffic-state records. They were retained in the audit table for review, but they were excluded from regular speed, travel-time, TTI, and CI calculation.

2. We also clarified that the public reproducibility package includes the data dictionary and API field-mapping notes. These files explain how the road-status field was interpreted and how closure-related records were separated from regular traffic-state records. We did not redistribute restricted raw API responses, API keys, or full vendor-returned geometries. Instead, we released cleaned and derived tables, scripts, and field explanations needed to reproduce the reported results.

3. For missing observations, we clarified that missing platform records were not interpolated. We made this choice because interpolation could make the panel appear more complete than it actually was. Missing records were treated as data-completeness issues, not as traffic-state values.

4. At the acquisition-code level, the workflow also includes scheduled completeness checks for each monitoring point. For example, if the returned records for point A02 are lower than the expected count within a checking window, the script re-queries the API for that point. The workflow then checks whether the newly returned record has already been saved in the database. This helps reduce missing acquisition results and avoids unnecessary duplicate database writing. This is an implementation-level acquisition and audit mechanism. We did not describe this example in detail in the main manuscript to avoid overloading the Methods section, but the released scripts, data dictionary, and API field-mapping notes document this acquisition and audit logic at the implementation level.

Change made: We added road-closure handling to Table 3 and added explanatory sentences in the Methods and Results. We also clarified that missing platform observations were not interpolated.

Revised manuscript text: "Store road-closure records in the audit table but exclude them from regular speed, travel-time, TTI, and CI calculation." (revised manuscript, Table 3)

Revised manuscript text: "Road-closure records are not deleted from the audit trail. They are stored separately so that abnormal operational events can be reviewed without mixing them with ordinary traffic-state estimates." (revised manuscript, lines 263--265)

Revised manuscript text: "Missing platform observations were not interpolated because interpolation could make the data appear more complete than they were. Road-closure records were retained in the audit trail but excluded from regular speed, travel-time, TTI, and CI calculation." (revised manuscript, lines 354--358)

**Reviewer #2 comment 9: Please reduce the impression that the paper is only a technical system description.**

Response: Thank you. We revised the writing to reduce the impression that the manuscript is only a technical system description. We changed the emphasis from software implementation to a corridor-monitoring research workflow. We also strengthened the research gap, research questions, contribution statement, benchmark interpretation, and deployment value. The revised manuscript now explains why the workflow is needed for data-scarce corridors, how it differs from previous FCD studies, and how the outputs should be interpreted as data completeness, traffic-state detection, and platform agreement rather than as a purely technical system output.

Change made: We revised the Introduction, Methods, Results, and Discussion to connect the workflow to the research gap, research questions, evidence boundary, and deployment value. We also reduced over-technical wording and used simpler sentences.

Revised manuscript text: To address these gaps, this study develops a script-based FCD workflow for continuous corridor monitoring. (revised manuscript, lines 43--44)

Revised manuscript text: The proposed workflow was designed to build a stable corridor traffic-data process. It collects platform traffic-state data, identifies invalid records, stores point-level records, and uses HERE data for comparison. (revised manuscript, lines 127--129)

Revised manuscript text: This study extends that work by combining data collection, field parsing, rule-based cleaning, audit logs, storage, indicator calculation, cross-platform comparison, and export in one workflow. (revised manuscript, lines 513--516)

Revised manuscript text: The workflow separates data completeness, traffic-state detection, and platform comparison. In conventional traffic-data analysis, these layers can be mixed: a complete dataset may be treated as a reliable traffic estimate, or a strong platform correlation may be described as validation. This study avoids that problem by treating daily availability as data completeness, TomTom indicators as descriptive traffic-state results, and TomTom--HERE agreement as platform agreement. (revised manuscript, lines 539--544)

**Reviewer #2 comment 10: Please strengthen the discussion of deployment value and future work.**

Response: Thank you. We revised the Discussion and Conclusion to make the deployment value and future work clearer. We now state that the proposed workflow is a supplementary screening method for data-scarce highway corridors. It provides a lightweight monitoring option before agencies invest in expensive roadside sensors. This is especially relevant for long-distance and remote corridors where continuous detector installation is costly and difficult to maintain. The workflow can help government agencies and transport departments identify high-pressure time windows, sections requiring field checking, and candidate locations for future permanent sensors. We also clarified that the workflow does not replace calibrated field detectors. It should be used as an initial screening and planning tool. Future work will add field validation, extend the TomTom--HERE benchmark period, include explanatory variables, and test the workflow on additional data-scarce corridors.

Change made: We added and revised Discussion paragraphs on deployment value, transferability, limitations, and future research.

Revised manuscript text: The workflow can provide a low-cost screening step before agencies invest in permanent sensors. (revised manuscript, lines 585--586)

Revised manuscript text: The proposed workflow should be used as a supplementary screening method, not as a replacement for calibrated field detectors. It can support screening of high-pressure periods, block-level diagnosis, field-check prioritization, and longer-term monitoring design where permanent sensors are limited. (revised manuscript, lines 666--668)

Revised manuscript text: Future work should add field validation, extend cross-platform benchmark sampling beyond the initial one-month HERE test, report block-specific and sensitivity outputs from the released matched table, connect explanatory variables such as rainfall, incidents, traffic volume, and road geometry, and test the workflow on additional data-scarce corridors. (revised manuscript, lines 671--675)

Revised manuscript text: The proposed workflow does not replace calibrated field detectors. Instead, it helps agencies identify high-pressure time windows, sections requiring field checking, and candidate locations for future permanent sensors. (revised manuscript, lines 589--592)

Revised manuscript text: The proposed workflow provides an initial monitoring layer for such conditions. It can support screening, temporal pattern recognition, field-validation prioritization, and longer-term monitoring design before agencies commit to more expensive sensing infrastructure. (revised manuscript, lines 605--608)

**Additional revisions made**

In addition to the changes above, we revised the language throughout the manuscript to use simpler sentences and to avoid over-claiming. We also updated the Data Availability Statement, Supporting Information, figure file names, and references.

**Closing statement**

We thank the Editor and reviewers again for their helpful comments. We believe that the revised manuscript is clearer about the data sources, the benchmark design, the limits of the TomTom--HERE comparison, the reproducibility package, and the value of the workflow for data-scarce highway corridors.

Sincerely,

Sun Wenjie, on behalf of all authors
