# Supplementary material for: Reproducible floating car data monitoring for data-scarce highway corridors: A TomTom–HERE benchmark study
Source: PLoS One. 2026 Aug 31;21(8):e0357158. doi: 10.1371/journal.pone.0357158 (PMC13529016; doi:10.1371/journal.pone.0357158)
Supplement: Supplementary file 3 [file pone.0357158.s009.docx]

**Response to the Academic Editor and Reviewers**

**Manuscript ID: PONE-D-26-21882R1**

Reproducible floating car data monitoring for data-scarce highway corridors: A TomTom-HERE benchmark study

**Journal:** PLOS ONE

**Corresponding author:** Sun Wenjie

**Revision type:** Point-by-point response to the minor-revision comments

# Dear Dr. Charitha Dias and Reviewers,

Thank you for the careful assessment of our revised manuscript. We have addressed every point raised in this review round. The revision focuses on four areas: clarifying the spatial design and monitoring-point selection; reporting the principal sensitivity-analysis results in the main Results section; reducing repeated evidence-boundary statements and moving detailed implementation information to Supporting Information; and distinguishing the proposed operational workflow from studies that only analyze a previously assembled FCD dataset.

All substantive changes are highlighted in the marked manuscript. The clean revised manuscript contains the same final text without highlighting. Our detailed responses are provided below.

**Summary of the revision**

**1. Spatial design and point selection:** Table 1 and the accompanying Methods text now report the geographic coverage, approximate length, HERE bounding-box coordinates, TomTom point assignments, and manual QGIS/OpenStreetMap review procedure.

**2. Sensitivity analyses:** The principal sensitivity-analysis results are now reported directly in the Results, while the complete sensitivity-analysis dataset has been uploaded to the public GitHub repository referenced in the Data Availability Statement. The Conclusion no longer describes these completed analyses as future work.

**3. Repetition and implementation detail:** Repeated statements about platform agreement and ground-truth validation were condensed, while detailed computing and logging information was moved to S6 Text.

**4. Workflow contribution:** The Introduction and Discussion now explicitly distinguish the study from retrospective analyses of precollected FCD datasets.

**Response to Reviewer #1**

**Reviewer comment:** Reviewer #1 did not request further revisions in this round.

**Response:** We thank Reviewer #1 for the positive assessment and for confirming that no additional revision was required. The manuscript changes described below were made in response to Reviewer #2 and were checked to ensure that they did not weaken the evidence boundaries and limitations established in the previous revision.

**Response to Reviewer #2**

**Comment 1. Spatial coverage, block length, and TomTom point selection**

**Reviewer comment:** Table 1 shows the point assignments, but the approximate length and geographic coverage of each HERE block are still missing. Please clarify whether the 42 TomTom points were initially equally spaced, selected near important locations, or manually adjusted on the basis of road matching.

**Response:** Thank you for identifying this remaining lack of spatial detail. We revised the spatial-design description and Table 1 as follows:

- Table 1 now reports the approximate geographic coverage, approximate corridor length, HERE bounding-box coordinates, assigned TomTom point IDs, and number of points for each block.
- Block 1 is described as the approximately 25 km Sematan-Telok Melano coastal section; Block 2 as the approximately 45 km Batu Kawa-Lundu-Sematan intermediate section; and Block 3 as the approximately 60 km Kuching-Batu Kawa urban-fringe section.
- The manuscript now explicitly states that the 42 TomTom locations were not designed as a strictly equally spaced statistical sample.
- The monitoring points were fixed, non-random anchors distributed to represent the main corridor environments while maintaining longitudinal coverage.
- All corridor alignments and monitoring-point locations were manually reviewed in QGIS 3.22.4 with reference to OpenStreetMap road data.
- Road alignments and point locations were adjusted where necessary to reduce ambiguous matching with nearby roads or junctions and to improve section-length calculation and block assignment.
- QGIS and OpenStreetMap were added as References 30 and 31.

**Changes made:** Materials and methods; Table 1; the paragraph following Table 1; References 30 and 31.

**Revised text:**

The 42 TomTom virtual monitoring points were selected to provide repeatable longitudinal coverage along the approximately 130 km corridor. They were not designed as a strictly equally spaced statistical sample.

All corridor alignments and monitoring-point locations were manually reviewed in QGIS 3.22.4 with reference to OpenStreetMap road data [30,31]. The road alignments and point locations were adjusted where necessary to maintain consistency with the intended highway corridor, reduce ambiguous matching with nearby roads or junctions, and improve the reliability of section-length calculation and block assignment.

**Comment 2. Numerical reporting of sensitivity analyses and Conclusion consistency**

**Reviewer comment:** The Results section refers readers to S5 Table, but the principal sensitivity results are not reported in the main text. The Conclusion also presents the already completed sensitivity analysis as future work.

**Response:** We agree that the principal robustness findings should be visible in the main article. We therefore added a numerical summary to the Results and corrected the Conclusion.

- Bootstrap resampling produced a pooled Pearson estimate of 0.919 with a 95% confidence interval of 0.915-0.933.
- The pooled Spearman estimate was 0.656 with a 95% confidence interval of 0.627-0.682.
- Across 24 daily strata, Pearson coefficients ranged from 0.810 to 0.975 and Spearman coefficients ranged from 0.501 to 0.762.
- For temporal shifts of -30, 0, and +30 min, the pooled Pearson coefficients were 0.921, 0.919, and 0.912, respectively; the corresponding Spearman coefficients were 0.645, 0.656, and 0.661.
- The pooled peak-overlap Jaccard indices were 0.566, 0.715, and 0.870 under the upper 10%, 15%, and 20% thresholds.
- Block 3 showed strong agreement (Pearson r = 0.910; Spearman rho = 0.898), whereas Blocks 1 and 2 showed weak or negative Pearson associations (r = -0.051 and -0.040).
- The Results and Discussion now emphasize that the pooled association coexists with substantial block-level heterogeneity.
- The Conclusion no longer lists the reporting of block-specific or sensitivity outputs as future work.

**Changes made:** Results; Discussion; Conclusion; and the complete sensitivity-analysis dataset deposited in the public GitHub repository referenced in the Data Availability Statement.

**Revised text:**

The principal sensitivity-analysis results are reported numerically in the Results section, while the complete sensitivity-analysis dataset is available in the public GitHub repository referenced in the Data Availability Statement.Bootstrap resampling of the released matched table produced a pooled Pearson estimate of 0.919 (95% CI: 0.915-0.933) and a pooled Spearman estimate of 0.656 (95% CI: 0.627-0.682).

Block-specific results were heterogeneous: Block 3 showed strong agreement, whereas Blocks 1 and 2 showed weak or negative associations.

Future work should add independent field measurements, extend the cross-platform overlap beyond the initial one-month HERE period, incorporate explanatory variables, and test the workflow on additional data-scarce corridors.

**Comment 3. Repetition and excessive implementation detail**

**Reviewer comment:** The distinction between platform agreement and ground-truth validation is repeated too often. The hardware and software description is also overly technical and should be shortened or moved to Supporting Information.

**Response:** We revised the manuscript to retain the necessary methodological qualification while removing repetitive formulations.

- Repeated statements about platform agreement and ground-truth validation were condensed across the Introduction, Materials and methods, Results, and Discussion.
- One clear evidence-boundary statement is retained where it is methodologically necessary.
- The main manuscript now reports only the functional implementation: Java and Spring Boot for acquisition, PostgreSQL for records and audit logs, and Python with pandas and NumPy for processing and figure reproduction.
- Detailed CPU, memory, storage, operating-system, GPU, Redis/cache, software-version, and task-level logging information was moved to S6 Text.
- The phrase 'Reviewer-requested robustness checks' was replaced with the neutral expression 'Sensitivity analysis of the matched benchmark.'

**Changes made:** Introduction; Materials and methods; Results; Discussion; Table 3; S6 Text.

**Revised text:**

The acquisition and analysis pipeline was implemented on a standard local workstation. Java and Spring Boot managed scheduled acquisition, PostgreSQL stored cleaned records and audit logs, and Python with pandas and NumPy supported cleaning, aggregation, indicator calculation, and figure reproduction [12-15]. Detailed hardware specifications, software versions, cache implementation, and task-level logging fields are provided in S6 Text.

The TomTom-HERE benchmark evaluates agreement between two vendor-processed signals rather than accuracy against an independent field reference.

**Comment 4. Distinction from studies using precollected FCD datasets**

**Reviewer comment:** Please strengthen the distinction between the proposed workflow and studies that only analyze an already collected or preassembled FCD dataset.

**Response:** We revised the Introduction and Discussion so that this methodological distinction is explicit.

- The Introduction now explains that the monitoring process was specified and operated before the final analytical dataset existed.
- The fixed coordinates, request schedule, response-field rules, missing-record logs, storage structure, spatial harmonization, and reproducible exports are treated as research-design components.
- The study evaluates both the operation and auditability of the data-producing system and the traffic patterns derived from the resulting records.
- The Discussion contrasts this design with retrospective analyses that begin with a completed FCD dataset and cannot fully audit acquisition failures, missing intervals, or spatial-matching decisions.
- The manuscript continues to describe the contribution as a reproducible research workflow rather than a general-purpose commercial software package.

**Changes made:** Introduction and Discussion.

**Revised text:**

Unlike studies that begin with an already assembled FCD dataset and proceed directly to traffic analysis, this study specified and operated the monitoring process before the final analytical dataset was produced.

The methodological contribution lies in treating FCD monitoring as a data-production and audit process rather than only as retrospective analysis of a completed dataset.

**Additional consistency checks**

- Table 1, the accompanying Methods text, and Fig 1 use the same three-block spatial design and the same TomTom point assignments.
- QGIS 3.22.4 and OpenStreetMap are cited in the main text and listed as References 30 and 31.
- The Conclusion summarizes the completed sensitivity analyses before stating genuine future research needs.
- The complete sensitivity-analysis dataset has been deposited in the public GitHub repository referenced in the Data Availability Statement. S6 Text is provided as Supporting Information.

**Closing statement**

We appreciate the reviewer's careful comments. The revisions have improved the transparency of the spatial design, the visibility and interpretation of the robustness analyses, the balance between methodological qualification and readability, and the articulation of the workflow contribution. We believe that the revised manuscript now addresses all comments raised in this review round.

Sincerely,

**Sun Wenjie, on behalf of all authors**

Corresponding author
