# Supplementary material for: Reproducible floating car data monitoring for data-scarce highway corridors: A TomTom–HERE benchmark study
Source: PLoS One. 2026 Aug 31;21(8):e0357158. doi: 10.1371/journal.pone.0357158 (PMC13529016; doi:10.1371/journal.pone.0357158)
Supplement: Peer review history

# Peer review: Decision Letter 0

**DOI:** 10.1371/journal.pone.0357158.r001

**Authors:** Charitha Dias

29 Jun 2026

Dear Dr. Sun,

Thank you for submitting your manuscript to PLOS ONE. After careful consideration, we feel that it has merit but does not fully meet PLOS ONE’s publication criteria as it currently stands. Therefore, we invite you to submit a revised version of the manuscript that addresses the points raised during the review process.

Please submit your revised manuscript by Aug 28 2026 11:59PM. If you will need more time than this to complete your revisions, please reply to this message or contact the journal office at plosone@plos.org. When you're ready to submit your revision, log on to https://www.editorialmanager.com/pone/ and select the 'Submissions Needing Revision' folder to locate your manuscript file.

- A letter that responds to each point raised by the academic editor and reviewer(s). You should upload this letter as a separate file labeled 'Response to Reviewers'.
- A marked-up copy of your manuscript that highlights changes made to the original version. You should upload this as a separate file labeled 'Revised Manuscript with Track Changes'.
- An unmarked version of your revised paper without tracked changes. You should upload this as a separate file labeled 'Manuscript'.

If you would like to make changes to your financial disclosure, please include your updated statement in your cover letter. Guidelines for resubmitting your figure files are available below the reviewer comments at the end of this letter.

If applicable, we recommend that you deposit your laboratory protocols in protocols.io to enhance the reproducibility of your results. Protocols.io assigns your protocol its own identifier (DOI) so that it can be cited independently in the future. For instructions see: https://journals.plos.org/plosone/s/submission-guidelines#loc-laboratory-protocols. Additionally, PLOS ONE offers an option for publishing peer-reviewed Lab Protocol articles, which describe protocols hosted on protocols.io. Read more information on sharing protocols at https://plos.org/protocols?utm_medium=editorial-email&utm_source=authorletters&utm_campaign=protocols.

As the corresponding author, your ORCID iD is verified in the submission system and will appear in the published article. PLOS supports the use of ORCID, and we encourage all coauthors to register for an ORCID iD and use it as well. Please encourage your coauthors to verify their ORCID iD within the submission system before final acceptance, as unverified ORCID iDs will not appear in the published article. *Only*  the individual author can complete the verification step; PLOS staff *cannot*  verify ORCID iDs on behalf of authors.

We look forward to receiving your revised manuscript.

Kind regards,

Charitha Dias

Academic Editor

PLOS One

Journal Requirements:

When submitting your revision, we need you to address these additional requirements.

1.Please ensure that your manuscript meets PLOS ONE's style requirements, including those for file naming. The PLOS ONE style templates can be found at

https://journals.plos.org/plosone/s/file?id=wjVg/PLOSOne_formatting_sample_main_body.pdf and

https://journals.plos.org/plosone/s/file?id=ba62/PLOSOne_formatting_sample_title_authors_affiliations.pdf

2. In your Methods section, please include additional information about your dataset and ensure that you have included a statement specifying whether the collection and analysis method complied with the terms and conditions for the source of the data.

3. We note that Figure 1 and 3 in your submission contain map images which may be copyrighted. All PLOS content is published under the Creative Commons Attribution License (CC BY 4.0), which means that the manuscript, images, and Supporting Information files will be freely available online, and any third party is permitted to access, download, copy, distribute, and use these materials in any way, even commercially, with proper attribution. For these reasons, we cannot publish previously copyrighted maps or satellite images created using proprietary data, such as Google software (Google Maps, Street View, and Earth). For more information, see our copyright guidelines: http://journals.plos.org/plosone/s/licenses-and-copyright.

We require you to either (1) present written permission from the copyright holder to publish these figures specifically under the CC BY 4.0 license, or (2) remove the figures from your submission:

1. You may seek permission from the original copyright holder of Figure 1 and 3 to publish the content specifically under the CC BY 4.0 license.

We recommend that you contact the original copyright holder with the Content Permission Form (http://journals.plos.org/plosone/s/file?id=7c09/content-permission-form.pdf) and the following text:

“I request permission for the open-access journal PLOS ONE to publish XXX under the Creative Commons Attribution License (CCAL) CC BY 4.0 (http://creativecommons.org/licenses/by/4.0/). Please be aware that this license allows unrestricted use and distribution, even commercially, by third parties. Please reply and provide explicit written permission to publish XXX under a CC BY license and complete the attached form.”

Please upload the completed Content Permission Form or other proof of granted permissions as an "Other" file with your submission.

In the figure caption of the copyrighted figure, please include the following text: “Reprinted from [ref] under a CC BY license, with permission from [name of publisher], original copyright [original copyright year].”

2. If you are unable to obtain permission from the original copyright holder to publish these figures under the CC BY 4.0 license or if the copyright holder’s requirements are incompatible with the CC BY 4.0 license, please either i) remove the figure or ii) supply a replacement figure that complies with the CC BY 4.0 license. Please check copyright information on all replacement figures and update the figure caption with source information. If applicable, please specify in the figure caption text when a figure is similar but not identical to the original image and is therefore for illustrative purposes only.

The following resources for replacing copyrighted map figures may be helpful:

USGS National Map Viewer (public domain): http://viewer.nationalmap.gov/viewer/

The Gateway to Astronaut Photography of Earth (public domain): http://eol.jsc.nasa.gov/sseop/clickmap/

Maps at the CIA (public domain): https://www.cia.gov/library/publications/the-world-factbook/index.html and https://www.cia.gov/library/publications/cia-maps-publications/index.html

NASA Earth Observatory (public domain): http://earthobservatory.nasa.gov/

Landsat: http://landsat.visibleearth.nasa.gov/

USGS EROS (Earth Resources Observatory and Science (EROS) Center) (public domain): http://eros.usgs.gov/#

Natural Earth (public domain): http://www.naturalearthdata.com/

4. If the reviewer comments include a recommendation to cite specific previously published works, please review and evaluate these publications to determine whether they are relevant and should be cited. There is no requirement to cite these works unless the editor has indicated otherwise.

Additional Editor Comments:

Two reviewers have completed their reviews and provided the comments below. Both of them particularly comments on validation methodology and limitations of the study.

[Note: HTML markup is below. Please do not edit.]

Reviewers' comments:

Reviewer's Responses to Questions

**Comments to the Author**

1. Is the manuscript technically sound, and do the data support the conclusions?

Reviewer #1: Partly

Reviewer #2: Yes

**********

2. Has the statistical analysis been performed appropriately and rigorously?

Reviewer #1: Yes

Reviewer #2: Yes

**********

3. Have the authors made all data underlying the findings in their manuscript fully available?

The PLOS Data policy

Reviewer #1: Yes

Reviewer #2: Yes

**********

4. Is the manuscript presented in an intelligible fashion and written in standard English?

Reviewer #1: Yes

Reviewer #2: Yes

**********

Reviewer #1: The paper is very interesting and relevant, given its focus on big data, traffic management, etc.

The authors mention the limitation of missing ground truth - however, one would expect that the potential implications are at least briefly introduced and discussed - What is general experience with probe data validation? How big differences in speed may be expected? Which differences may be between TomTom and HERE data - and why? I recommend adding these points to the paper.

After this update, the paper should be re-submitted and re-reviewed.

Reviewer #2: The manuscript addresses a relevant and timely topic in traffic monitoring, particularly for highway corridors where fixed sensing infrastructure is limited. However, several aspects require further strengthening.

Major comments:

Q1. Explain more explicitly what is new compared with existing FCD monitoring frameworks.

Q2. The authors appropriately state that HERE is not ground truth. However, could the manuscript further discuss the possibility that TomTom and HERE are not fully independent data sources?

Q3. The HERE benchmark period is much shorter than the TomTom monitoring period. This limitation should be made more visible in the results and conclusion so that readers do not interpret the benchmark findings as representing the full monitoring period.

Q4. The use of Pearson and Spearman correlations is useful, but the matched data consist of repeated block–time observations. Could the authors consider adding daily stratified correlations, confidence intervals, or cluster-aware sensitivity checks to account for temporal and block-level dependence?

Q5. How were the 42 TomTom virtual monitoring points selected along the 130 km corridor? In addition, explain how the three HERE benchmark blocks were defined and how TomTom points and HERE segments were assigned to each block. A short table would improve.

Minor comments:

Q1. The phrase “data-scarce corridors” is central to the paper. Define this term more formally in the introduction.

Q2. Some sentences are slightly repetitive, especially in the introduction and discussion where the manuscript repeatedly states that the study is methodological and not a ground-truth validation.

Q3. Clarify whether road-closure records were fully excluded or separately stored for operational interpretation.

Q4. The paper mentions sensitivity checks but the main results do not clearly report these outputs.

Q5. Several sections read more like a technical system description than a research article.

**********

what does this mean?). If published, this will include your full peer review and any attached files.

If you choose “no”, your identity will remain anonymous but your review may still be made public.

**Do you want your identity to be public for this peer review?**  For information about this choice, including consent withdrawal, please see our Privacy Policy

Reviewer #1: No

Reviewer #2: No

**********

[NOTE: If reviewer comments were submitted as an attachment file, they will be attached to this email and accessible via the submission site. Please log into your account, locate the manuscript record, and check for the action link "View Attachments". If this link does not appear, there are no attachment files.]

To ensure your figures meet our technical requirements, please review our figure guidelines: https://journals.plos.org/plosone/s/figures

You may also use PLOS’s free figure tool, NAAS, to help you prepare publication quality figures: https://journals.plos.org/plosone/s/figures#loc-tools-for-figure-preparation.

NAAS will assess whether your figures meet our technical requirements by comparing each figure against our figure specifications.

# Author comment: Author response to Decision Letter 1

**DOI:** 10.1371/journal.pone.0357158.r002

2 Jul 2026

Dear Editor and Reviewers,

Thank you for the opportunity to revise our manuscript entitled "Reproducible floating car data monitoring for data-scarce highway corridors: A TomTom--HERE benchmark study" (PONE-D-26-21882).

We have uploaded a detailed point-by-point response to the Editor and reviewers as a separate file. In the revised manuscript, we clarified the TomTom and HERE data sources and redistribution conditions, addressed the map-copyright issue by using public-domain Natural Earth basemap data and revising the previous map-based Fig 3, strengthened the explanation of the 42 TomTom monitoring points and three HERE benchmark blocks, clarified that the TomTom--HERE comparison is platform agreement rather than independent ground-truth validation, added sensitivity-check information, explained the handling of road-closure records and missing observations, and expanded the discussion of deployment value and future work.

We have also uploaded the clean revised manuscript, highlighted revised manuscript, response letter, and separate revised figure files.

Thank you again for your time and constructive comments.

Sincerely,

Wenjie Sun

On behalf of all authors

Attachment Submitted filename: Response_to_Reviewers_PLOS_ONE.docx

# Peer review: Decision Letter 1

**DOI:** 10.1371/journal.pone.0357158.r003

**Authors:** Charitha Dias

3 Aug 2026

Dear Dr. Sun,

Thank you for submitting your manuscript to PLOS ONE. After careful consideration, we feel that it has merit but does not fully meet PLOS ONE’s publication criteria as it currently stands. Therefore, we invite you to submit a revised version of the manuscript that addresses the points raised during the review process.

Please submit your revised manuscript by Sep 02 2026 11:59PM. If you will need more time than this to complete your revisions, please reply to this message or contact the journal office at plosone@plos.org. When you're ready to submit your revision, log on to https://www.editorialmanager.com/pone/ and select the 'Submissions Needing Revision' folder to locate your manuscript file.

Please include the following items when submitting your revised manuscript:

- A letter that responds to each point raised by the academic editor and reviewer(s). You should upload this letter as a separate file labeled 'Response to Reviewers'.
- A marked-up copy of your manuscript that highlights changes made to the original version. You should upload this as a separate file labeled 'Revised Manuscript with Track Changes'.
- An unmarked version of your revised paper without tracked changes. You should upload this as a separate file labeled 'Manuscript'.

If you would like to make changes to your financial disclosure, please include your updated statement in your cover letter. Guidelines for resubmitting your figure files are available below the reviewer comments at the end of this letter.

If applicable, we recommend that you deposit your laboratory protocols in protocols.io to enhance the reproducibility of your results. Protocols.io assigns your protocol its own identifier (DOI) so that it can be cited independently in the future. For instructions see: https://journals.plos.org/plosone/s/submission-guidelines#loc-laboratory-protocols. Additionally, PLOS ONE offers an option for publishing peer-reviewed Lab Protocol articles, which describe protocols hosted on protocols.io. Read more information on sharing protocols at https://plos.org/protocols?utm_medium=editorial-email&utm_source=authorletters&utm_campaign=protocols.

As the corresponding author, your ORCID iD is verified in the submission system and will appear in the published article. PLOS supports the use of ORCID, and we encourage all coauthors to register for an ORCID iD and use it as well. Please encourage your coauthors to verify their ORCID iD within the submission system before final acceptance, as unverified ORCID iDs will not appear in the published article. *Only*  the individual author can complete the verification step; PLOS staff *cannot*  verify ORCID iDs on behalf of authors.

We look forward to receiving your revised manuscript.

Kind regards,

Charitha Dias

Academic Editor

PLOS One

Journal Requirements:

If the reviewer comments include a recommendation to cite specific previously published works, please review and evaluate these publications to determine whether they are relevant and should be cited. There is no requirement to cite these works unless the editor has indicated otherwise.

Please review your reference list to ensure that it is complete and correct. If you have cited papers that have been retracted, please include the rationale for doing so in the manuscript text, or remove these references and replace them with relevant current references. Any changes to the reference list should be mentioned in the rebuttal letter that accompanies your revised manuscript. If you need to cite a retracted article, indicate the article’s retracted status in the References list and also include a citation and full reference for the retraction notice.

**Additional Editor Comments:**

Although the reviewers were satisfied with the authors' responses and the revised manuscript, one reviewer raised a few minor comments.

[Note: HTML markup is below. Please do not edit.]

Reviewers' comments:

Reviewer's Responses to Questions

**Comments to the Author**

Reviewer #1: All comments have been addressed

Reviewer #2: (No Response)

**********

2. Is the manuscript technically sound, and do the data support the conclusions?

Reviewer #1: Yes

Reviewer #2: Yes

**********

3. Has the statistical analysis been performed appropriately and rigorously?

Reviewer #1: Yes

Reviewer #2: Yes

**********

4. Have the authors made all data underlying the findings in their manuscript fully available?

The PLOS Data policy

Reviewer #1: Yes

Reviewer #2: Yes

**********

5. Is the manuscript presented in an intelligible fashion and written in standard English?

Reviewer #1: Yes

Reviewer #2: Yes

**********

Reviewer #1: Thank you for the revision. I can see that the review comments were addressed and the paper quality has improved. I recommend the paper to be accepted for publication in the journal.

Reviewer #2: The authors have addressed most of the previous comments, but a few issues still require attention.

Q1. Table 1 now shows the point assignments, but the approximate length and geographic coverage of each HERE block are still missing. Clarify whether the 42 TomTom points were initially equally spaced, selected near important locations, or manually adjusted based on road matching.

Q2. The Results state that the sensitivity checks are reported in S5 Table, while the Conclusion still presents them as future work. Please correct this inconsistency and briefly report the main numerical findings in the Results.

Q3. The distinction between platform agreement and ground-truth validation is repeated too often. Some hardware and software implementation details also remain overly technical and could be shortened or moved to the Supporting Information.

Q4. State how the proposed workflow differs from studies that only analyze previously collected FCD datasets.

**********

what does this mean?). If published, this will include your full peer review and any attached files.

If you choose “no”, your identity will remain anonymous but your review may still be made public.

**Do you want your identity to be public for this peer review?**  For information about this choice, including consent withdrawal, please see our Privacy Policy

Reviewer #1: No

Reviewer #2: **Yes:** Siti Raudhatul Fadilah

**********

[NOTE: If reviewer comments were submitted as an attachment file, they will be attached to this email and accessible via the submission site. Please log into your account, locate the manuscript record, and check for the action link "View Attachments". If this link does not appear, there are no attachment files.]

To ensure your figures meet our technical requirements, please review our figure guidelines: https://journals.plos.org/plosone/s/figures

You may also use PLOS’s free figure tool, NAAS, to help you prepare publication quality figures: https://journals.plos.org/plosone/s/figures#loc-tools-for-figure-preparation.

NAAS will assess whether your figures meet our technical requirements by comparing each figure against our figure specifications.

# Author comment: Author response to Decision Letter 2

**DOI:** 10.1371/journal.pone.0357158.r004

5 Aug 2026

Summary of the revision

1. Spatial design and point selection: Table 1 and the accompanying Methods text now report the geographic coverage, approximate length, HERE bounding-box coordinates, TomTom point assignments, and manual QGIS/OpenStreetMap review procedure.

2. Sensitivity analyses: The principal sensitivity-analysis results are now reported directly in the Results, while the complete sensitivity-analysis dataset has been uploaded to the public GitHub repository referenced in the Data Availability Statement. The Conclusion no longer describes these completed analyses as future work.

3. Repetition and implementation detail: Repeated statements about platform agreement and ground-truth validation were condensed, while detailed computing and logging information was moved to S6 Text.

4. Workflow contribution: The Introduction and Discussion now explicitly distinguish the study from retrospective analyses of precollected FCD datasets.

Response to Reviewer #1

Reviewer comment: Reviewer #1 did not request further revisions in this round.

Response: We thank Reviewer #1 for the positive assessment and for confirming that no additional revision was required. The manuscript changes described below were made in response to Reviewer #2 and were checked to ensure that they did not weaken the evidence boundaries and limitations established in the previous revision.

Response to Reviewer #2

Comment 1. Spatial coverage, block length, and TomTom point selection

Reviewer comment: Table 1 shows the point assignments, but the approximate length and geographic coverage of each HERE block are still missing. Please clarify whether the 42 TomTom points were initially equally spaced, selected near important locations, or manually adjusted on the basis of road matching.

Response: Thank you for identifying this remaining lack of spatial detail. We revised the spatial-design description and Table 1 as follows:

�Table 1 now reports the approximate geographic coverage, approximate corridor length, HERE bounding-box coordinates, assigned TomTom point IDs, and number of points for each block.

�Block 1 is described as the approximately 25 km Sematan-Telok Melano coastal section; Block 2 as the approximately 45 km Batu Kawa-Lundu-Sematan intermediate section; and Block 3 as the approximately 60 km Kuching-Batu Kawa urban-fringe section.

�The manuscript now explicitly states that the 42 TomTom locations were not designed as a strictly equally spaced statistical sample.

�The monitoring points were fixed, non-random anchors distributed to represent the main corridor environments while maintaining longitudinal coverage.

�All corridor alignments and monitoring-point locations were manually reviewed in QGIS 3.22.4 with reference to OpenStreetMap road data.

�Road alignments and point locations were adjusted where necessary to reduce ambiguous matching with nearby roads or junctions and to improve section-length calculation and block assignment.

�QGIS and OpenStreetMap were added as References 30 and 31.

Changes made: Materials and methods; Table 1; the paragraph following Table 1; References 30 and 31.

Revised text:

The 42 TomTom virtual monitoring points were selected to provide repeatable longitudinal coverage along the approximately 130 km corridor. They were not designed as a strictly equally spaced statistical sample.

All corridor alignments and monitoring-point locations were manually reviewed in QGIS 3.22.4 with reference to OpenStreetMap road data [30,31]. The road alignments and point locations were adjusted where necessary to maintain consistency with the intended highway corridor, reduce ambiguous matching with nearby roads or junctions, and improve the reliability of section-length calculation and block assignment.

Comment 2. Numerical reporting of sensitivity analyses and Conclusion consistency

Reviewer comment: The Results section refers readers to S5 Table, but the principal sensitivity results are not reported in the main text. The Conclusion also presents the already completed sensitivity analysis as future work.

Response: We agree that the principal robustness findings should be visible in the main article. We therefore added a numerical summary to the Results and corrected the Conclusion.

�Bootstrap resampling produced a pooled Pearson estimate of 0.919 with a 95% confidence interval of 0.915-0.933.

�The pooled Spearman estimate was 0.656 with a 95% confidence interval of 0.627-0.682.

�Across 24 daily strata, Pearson coefficients ranged from 0.810 to 0.975 and Spearman coefficients ranged from 0.501 to 0.762.

�For temporal shifts of -30, 0, and +30 min, the pooled Pearson coefficients were 0.921, 0.919, and 0.912, respectively; the corresponding Spearman coefficients were 0.645, 0.656, and 0.661.

�The pooled peak-overlap Jaccard indices were 0.566, 0.715, and 0.870 under the upper 10%, 15%, and 20% thresholds.

�Block 3 showed strong agreement (Pearson r = 0.910; Spearman rho = 0.898), whereas Blocks 1 and 2 showed weak or negative Pearson associations (r = -0.051 and -0.040).

�The Results and Discussion now emphasize that the pooled association coexists with substantial block-level heterogeneity.

�The Conclusion no longer lists the reporting of block-specific or sensitivity outputs as future work.

Changes made: Results; Discussion; Conclusion; and the complete sensitivity-analysis dataset deposited in the public GitHub repository referenced in the Data Availability Statement.

Revised text:

The principal sensitivity-analysis results are reported numerically in the Results section, while the complete sensitivity-analysis dataset is available in the public GitHub repository referenced in the Data Availability Statement.Bootstrap resampling of the released matched table produced a pooled Pearson estimate of 0.919 (95% CI: 0.915-0.933) and a pooled Spearman estimate of 0.656 (95% CI: 0.627-0.682).

Block-specific results were heterogeneous: Block 3 showed strong agreement, whereas Blocks 1 and 2 showed weak or negative associations.

Future work should add independent field measurements, extend the cross-platform overlap beyond the initial one-month HERE period, incorporate explanatory variables, and test the workflow on additional data-scarce corridors.

Comment 3. Repetition and excessive implementation detail

Reviewer comment: The distinction between platform agreement and ground-truth validation is repeated too often. The hardware and software description is also overly technical and should be shortened or moved to Supporting Information.

Response: We revised the manuscript to retain the necessary methodological qualification while removing repetitive formulations.

�Repeated statements about platform agreement and ground-truth validation were condensed across the Introduction, Materials and methods, Results, and Discussion.

�One clear evidence-boundary statement is retained where it is methodologically necessary.

�The main manuscript now reports only the functional implementation: Java and Spring Boot for acquisition, PostgreSQL for records and audit logs, and Python with pandas and NumPy for processing and figure reproduction.

�Detailed CPU, memory, storage, operating-system, GPU, Redis/cache, software-version, and task-level logging information was moved to S6 Text.

�The phrase 'Reviewer-requested robustness checks' was replaced with the neutral expression 'Sensitivity analysis of the matched benchmark.'

Changes made: Introduction; Materials and methods; Results; Discussion; Table 3; S6 Text.

Revised text:

The acquisition and analysis pipeline was implemented on a standard local workstation. Java and Spring Boot managed scheduled acquisition, PostgreSQL stored cleaned records and audit logs, and Python with pandas and NumPy supported cleaning, aggregation, indicator calculation, and figure reproduction [12-15]. Detailed hardware specifications, software versions, cache implementation, and task-level logging fields are provided in S6 Text.

The TomTom-HERE benchmark evaluates agreement between two vendor-processed signals rather than accuracy against an independent field reference.

Comment 4. Distinction from studies using precollected FCD datasets

Reviewer comment: Please strengthen the distinction between the proposed workflow and studies that only analyze an already collected or preassembled FCD dataset.

Response: We revised the Introduction and Discussion so that this methodological distinction is explicit.

�The Introduction now explains that the monitoring process was specified and operated before the final analytical dataset existed.

�The fixed coordinates, request schedule, response-field rules, missing-record logs, storage structure, spatial harmonization, and reproducible exports are treated as research-design components.

�The study evaluates both the operation and auditability of the data-producing system and the traffic patterns derived from the resulting records.

�The Discussion contrasts this design with retrospective analyses that begin with a completed FCD dataset and cannot fully audit acquisition failures, missing intervals, or spatial-matching decisions.

�The manuscript continues to describe the contribution as a reproducible research workflow rather than a general-purpose commercial software package.

Changes made: Introduction and Discussion.

Revised text:

Unlike studies that begin with an already assembled FCD dataset and proceed directly to traffic analysis, this study specified and operated the monitoring process before the final analytical dataset was produced.

The methodological contribution lies in treating FCD monitoring as a data-production and audit process rather than only as retrospective analysis of a completed dataset.

Additional consistency checks

�Table 1, the accompanying Methods text, and Fig 1 use the same three-block spatial design and the same TomTom point assignments.

�QGIS 3.22.4 and OpenStreetMap are cited in the main text and listed as References 30 and 31.

�The Conclusion summarizes the completed sensitivity analyses before stating genuine future research needs.

�The complete sensitivity-analysis dataset has been deposited in the public GitHub repository referenced in the Data Availability Statement. S6 Text is provided as Supporting Information.

Closing statement

We appreciate the reviewer's careful comments. The revisions have improved the transparency of the spatial design, the visibility and interpretation of the robustness analyses, the balance between methodological qualification and readability, and the articulation of the workflow contribution. We believe that the revised manuscript now addresses all comments raised in this review round.

Sincerely,

Sun Wenjie, on behalf of all authors

Corresponding author

Attachment Submitted filename: 03_PONE-D-26-21882R1_Response_to_Reviewers.docx

# Peer review: Decision Letter 2

**DOI:** 10.1371/journal.pone.0357158.r005

**Authors:** Charitha Dias

12 Aug 2026

Reproducible floating car data monitoring for data-scarce highway corridors: A TomTom–HERE benchmark study

PONE-D-26-21882R2

Dear Dr. Sun,

We’re pleased to inform you that your manuscript has been judged scientifically suitable for publication and will be formally accepted for publication once it meets all outstanding technical requirements.

Within one week, you’ll receive an e-mail detailing the required amendments. When these have been addressed, you’ll receive a formal acceptance letter and your manuscript will be scheduled for publication.

An invoice will be generated when your article is formally accepted. Please note, if your institution has a publishing partnership with PLOS and your article meets the relevant criteria, all or part of your publication costs will be covered. Please make sure your user information is up-to-date by logging into Editorial Manager at Editorial Manager® and clicking the ‘Update My Information' link at the top of the page. For questions related to billing, please contact billing support.

If your institution or institutions have a press office, please notify them about your upcoming paper to help maximize its impact. If they’ll be preparing press materials, please inform our press team as soon as possible -- no later than 48 hours after receiving the formal acceptance. Your manuscript will remain under strict press embargo until 2 pm Eastern Time on the date of publication. For more information, please contact onepress@plos.org.

Kind regards,

Charitha Dias

Academic Editor

PLOS One

Additional Editor Comments (optional):

Reviewers' comments:

Reviewer's Responses to Questions

**Comments to the Author**

Reviewer #2: All comments have been addressed

**********

2. Is the manuscript technically sound, and do the data support the conclusions?

Reviewer #2: Yes

**********

3. Has the statistical analysis been performed appropriately and rigorously?

Reviewer #2: Yes

**********

4. Have the authors made all data underlying the findings in their manuscript fully available?

The PLOS Data policy

Reviewer #2: Yes

**********

5. Is the manuscript presented in an intelligible fashion and written in standard English?

Reviewer #2: Yes

**********

Reviewer #2: The authors have addressed my previous comments and revised the manuscript accordingly. I have no further concerns and recommend acceptance in its current form.

**********

what does this mean?). If published, this will include your full peer review and any attached files.

If you choose “no”, your identity will remain anonymous but your review may still be made public.

**Do you want your identity to be public for this peer review?**  For information about this choice, including consent withdrawal, please see our Privacy Policy

Reviewer #2: **Yes:** Siti Raudhatul Fadilah

**********

# Editor report: Acceptance letter

**DOI:** 10.1371/journal.pone.0357158.r006

**Authors:** Charitha Dias

PONE-D-26-21882R2

PLOS One

Dear Dr. Sun,

I'm pleased to inform you that your manuscript has been deemed suitable for publication in PLOS One. Congratulations! Your manuscript is now being handed over to our production team.

At this stage, our production department will prepare your paper for publication. This includes ensuring the following:

* All references, tables, and figures are properly cited

* All relevant supporting information is included in the manuscript submission,

* There are no issues that prevent the paper from being properly typeset

You will receive further instructions from the production team, including instructions on how to review your proof when it is ready. Please keep in mind that we are working through a large volume of accepted articles, so please give us a few days to review your paper and let you know the next and final steps.

Lastly, if your institution or institutions have a press office, please let them know about your upcoming paper now to help maximize its impact. If they'll be preparing press materials, please inform our press team within the next 48 hours. Your manuscript will remain under strict press embargo until 2 pm Eastern Time on the date of publication. For more information, please contact onepress@plos.org.

You will receive an invoice from PLOS for your publication fee after your manuscript has reached the completed accept phase. If you receive an email requesting payment before acceptance or for any other service, this may be a phishing scheme. Learn how to identify phishing emails and protect your accounts at https://explore.plos.org/phishing.

If we can help with anything else, please email us at customercare@plos.org.

Thank you for submitting your work to PLOS One and supporting open access.

Kind regards,

PLOS ONE Editorial Office Staff

on behalf of

Dr. Charitha Dias

Academic Editor

PLOS One
